# Interactions and Stability of Gut Microbiota in Zebrafish Increase with Host Development

**DOI:** 10.1128/spectrum.01696-21

**Published:** 2022-03-21

**Authors:** Fanshu Xiao, Wengen Zhu, Yuhe Yu, Jie Huang, Juan Li, Zhili He, Jianjun Wang, Huaqun Yin, Huang Yu, Shengwei Liu, Pubo Chen, Zhijian Huang, Jianguo He, Cheng Wang, Longfei Shu, Qingyun Yan

**Affiliations:** a Center for Precision Medicine, Medical Research Center, Guangdong Provincial People’s Hospital, Guangdong Academy of Medical Sciences, Guangzhou, China; b Environmental Microbiomics Research Center, School of Environmental Science and Engineering, Southern Marine Science and Engineering Guangdong Laboratory (Zhuhai), State Key Laboratory for Biocontrol, Sun Yat-sen University, Guangzhou, China; c Key Laboratory of Aquatic Biodiversity and Conservation of Chinese Academy of Sciences, Institute of Hydrobiology, Chinese Academy of Sciences, Wuhan, China; d College of Agronomy, Hunan Agricultural University, Changsha, China; e Nanjing Institute of Geography and Limnology, Chinese Academy of Sciences, Nanjing, China; f School of Minerals Processing and Bioengineering, Central South University, Changsha, China; Temasek Life Sciences Laboratory

**Keywords:** zebrafish, gut microbiota, microbial interactions, ecosystem stability, keystone taxa

## Abstract

Understanding interactions within the gut microbiome and its stability are of critical importance for deciphering ecological issues within the gut ecosystem. Recent studies indicate that long-term instability of gut microbiota is associated with human diseases, and recovery of stability is helpful in the return to health. However, much less is known about such topics in fish, which encompass nearly half of all vertebrate diversity. Here, we examined the assembly and succession of gut microbiota in more than 550 zebrafish, and evaluated the variations of microbial interactions and stability across fish development from larva to adult using molecular ecological network analysis. We found that microbial interactions and stability in the fish gut ecosystem generally increased with host development. This could be attributed to the development of the zebrafish immune system, the increasing amount of space available for microbial colonization within the gut, and the greater stability of nutrients available for the colonized microbiota in adult zebrafish. Moreover, the potential keystone taxa, even those with relatively low abundances, played important roles in affecting the microbial interactions and stability. These findings indicate that regulating rare keystone taxa in adult fish may have great potential in gut microbial management to maintain gut ecosystem stability, which could also provide references for managing gut microbiota in humans and other animals.

**IMPORTANCE** Understanding gut microbial stability and the underlying mechanisms is an important but largely ignored ecological issue in vertebrate fish. Here, using a zebrafish model and network analysis of the gut microbiota we found that microbial interactions and stability in the gut ecosystem increase with fish development. This finding has important implications for microbial management to maintain gut homeostasis and provide better gut ecosystem services for the host. First, future studies should always consider using fish of different age groups to gain a full understanding of gut microbial networks. Second, management of the keystone taxa, even those that are only present at a low abundance, during the adult stage may be a viable pathway to maintain gut ecosystem stability. This study greatly expands our current knowledge regarding gut ecosystem stability in terms of ecological networks affected by fish development, and also highlights potential directions for gut microbial management in humans and other animals.

## INTRODUCTION

Each fish gut ecosystem is colonized by a diverse community of microorganisms from the surrounding environment during food consumption (1, 2). Mounting evidence has indicated that the composition and structure of gut microbiota in fish can vary considerably across host development (3–5), which also could be affected by numerous other factors including host immunity (6), diseases (7), diets (8), and the local environment (9). An increasing number of studies have indicated that both deterministic and neutral processes could affect the colonization of fish gut microbiota (4, 10), which play important roles in regulating the host’s functions. For example, gut microbiota in fish have been shown to be closely involved in regulating host metabolism (11), promoting growth (12), stimulating the immune system (13), affecting tissue development (14), and defense against some diseases (7). Unfortunately, how microorganisms that have colonized the fish gut ecosystem interact with each other, and the mechanism(s) regulating gut ecosystem stability remain unknown.

Generally, microbial taxa in a community depend on each other through a complex system of interconnections (15), which can be represented as ecological networks with species as nodes and their correlations as links (16). Recently, there has been increasing interest in the use of molecular ecological networks for characterizing the interactions of microbial communities. Moreover, microbial networks also provide information to identify potential keystone taxa (17) that greatly impact the structure and function of ecosystems, and thereby generate hypotheses for future experimental validation (18). Additionally, the complexity of interactions as visualized by the ecological networks could reflect the ecosystem stability (19), especially in food web-based networks (20). Intestinal *Bacteroides* have even shown species-specific interactions with the host and mediation of gut ecosystem stability (21). More recently, network-based stability of the human gut microbiome has been extended to address diseases-related issues. For example, Chen et al. (22) found that a human gut microbiome with higher diversity was more stable and better for host health, whereas long-term (e.g., 5 years) instability within the gut microbiome was associated with metabolic liver diseases (23). There is increasing evidence that a gut microbiome with an unbalanced or unhealthy stable state might lead to human diseases (24). However, we still lack a general understanding of whether and how the ecological networks of fish gut microbiota and their stability varies across host development.

**FIG 1:**
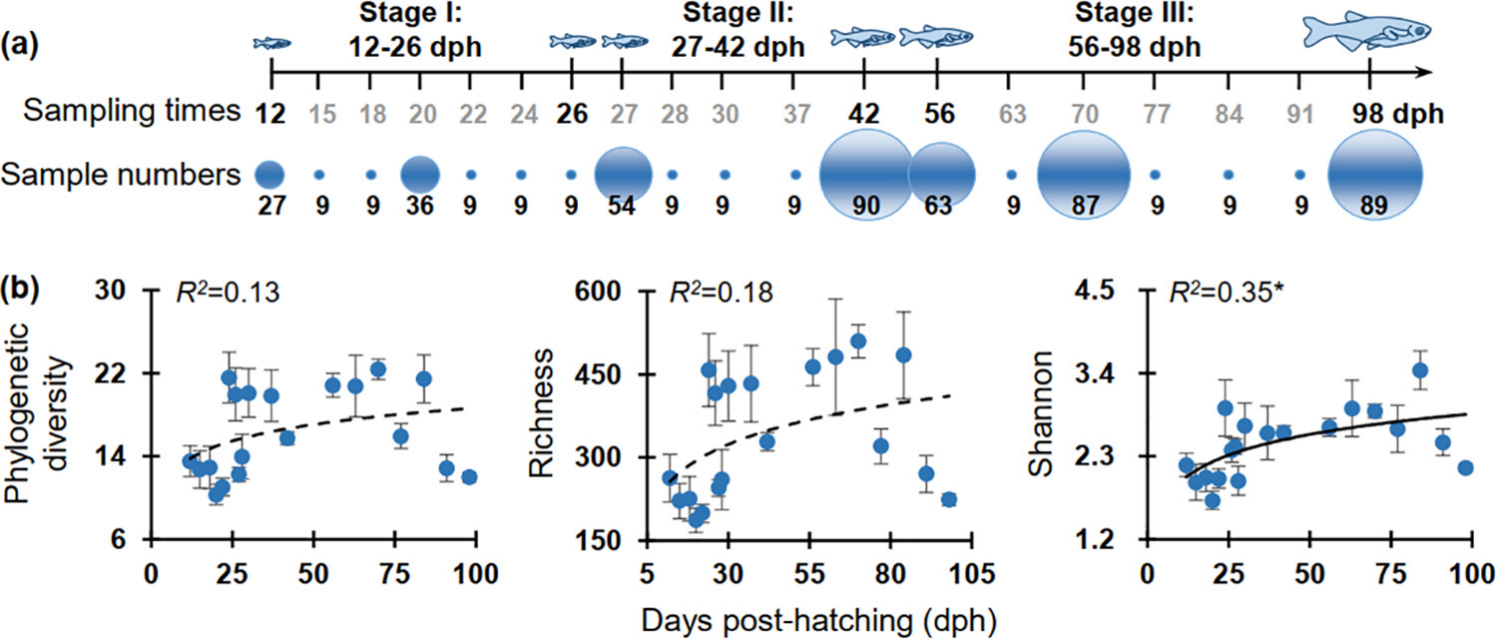
Sampling design and the diversity patterns of gut microbiota across zebrafish development from 12 to 98 days post-hatching (dph), which was divided into three stages as referred to in our previous study (5) according to the community patterns of gut microbiota. (a) Number of zebrafish individuals collected as replicates for each sampling point. To decrease the possible effects of diets, which changed more frequently before 12 dph, we chose the 12 dph as the first sampling point to analyze zebrafish gut microbiota. The intervals for most sampling points were 1 week, and different intervals (1 to 14 days) were also applied occasionally to address gut microbial variations within different days across zebrafish development. At each sampling point, we randomly selected at least three zebrafish individuals from each tank (i.e., 9 replicates from 3 tanks). However, to visualize the interactions and stability of gut microbiota by ecological network analysis, we increased the replicates from 27 to 90 at the sampling points of 12, 20, 27, 42, 56, 70, and 98 dph. Many more but different replicates were applied for these seven sampling points to decrease the possible sample effects involved in the network analysis. (b) Alpha diversity succession as visualized by the sampling points, and the adjusted *R*^2^ are given together with the corresponding *P* values (* 0.01 < *P < *0.05).

Using a powerful zebrafish model that was recently involved in studying host-microbial interactions (25, 26) and environmental effects on the gut microbiota (5), the current study highlights the importance of gut microbial network construction to evaluate fish gut microbial ecosystem stability across host development. As fish gut microbial diversity (5, 27), and the ecological driving processes (4, 10, 28) all closely correlate with host development, they may also have profound influences on the network complexity and stability of the fish gut ecosystem. We hypothesized that the gut microbial stability would increase with fish development due to enhanced microbial interactions and more stabilized nutrient niches in adult fish. To test this core hypothesis, we characterized the gut microbial networks across zebrafish development (in terms of both developmental stages and specific sampling points, Fig. 1) and identified potential keystone taxa that may considerably affect the assemblage pattern and stability of gut microbiota. We found that the microbial interactions and stability of gut microbiota in zebrafish increased from larval to adult stages, which greatly expands our current knowledge regarding how gut ecosystem stability is affected by host development. This finding highlights potential directions for gut microbial management to promote fish growth and defense against diseases.

## RESULTS

### Stage-dependent diversity patterns of gut microbiota across zebrafish development.

The amplified 16S rRNA gene was sequenced to visualize the diversity patterns of gut microbiota across zebrafish development from 12 to 98 days post-hatching (dph) (Fig. 1a). Analysis of the entire intestinal tract of each zebrafish indicated that both phylogenetic diversity and richness showed no clear temporal patterns over the 19 sampling points, and only Shannon diversity had a weak correlation (*R^2^* = 0.35, *P = *0.03) with zebrafish development (Fig. 1b). The dissimilarity tests based on Bray-Curtis and Jaccard distances of gut microbiota between each pair of adjacent sampling points also showed no significant difference (MRPP or PERMANOVA, *P > *0.05) for early sampling points (i.e., 12 to 18 dph), as well as during 28 to 37 dph, 56 to 63 dph, and 70 to 77 dph (data not shown). However, those among 12, 20, 27, 42, 56, 70, and 98 dph were always significantly different (MRPP or PERMANOVA, *P = *0.001, Table S1). Also, we observed similar diversity results if only the minimum number of samples (i.e., nine) were retained in the dissimilarity tests for all of these sampling points (data not shown).

### Stage-dependent characteristics of the constructed networks.

**FIG 2:**
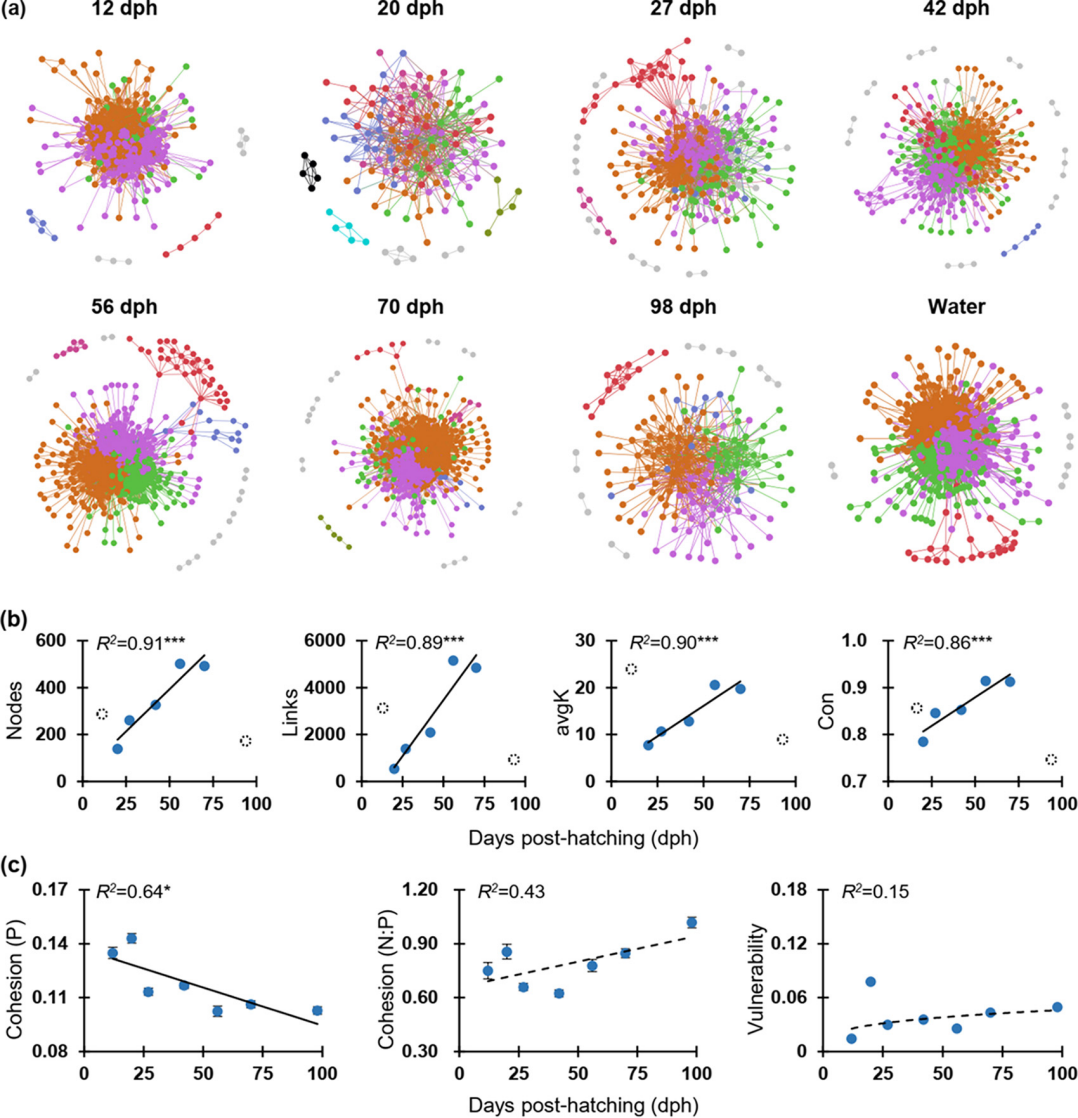
Succession and stability of gut microbial networks across zebrafish development (only for sampling points with ≥ 27 replicates). (a) Visualization of constructed molecular ecological networks generated using the Molecular Ecological Network Analysis (MENA) pipeline based on OTU relative abundances of gut microbiota. Each node represents 1 OTU, and each link represents a correlation between a pair of nodes. Large network modules (≥ 5 nodes) are shown in different colors, and smaller modules (2 to 4 nodes) are shown in gray. Details of network topological attributes are listed in Table S2. (b) Development-dependent changes of network topology included nodes, links, average degree (avgK), and connectedness (Con). In each panel, filled symbols represent networks involved in the significant (*P < *0.05) linear regression as shown by the solid line (including 20, 27, 42, 56, and 70 dph), and dotted open symbols represent those that were non-significant (*P > *0.05). The adjusted *R^2^* are given together with the corresponding *P* values (*** *P < *0.001). (c) Network stability, as visualized by sampling points, and the adjusted *R*^2^ are given together with the corresponding *P* values (solid lines: * 0.01 < *P < *0.05; dotted lines: *P > *0.05). The positive cohesion (P) and negative cohesion (N) reflect the magnitude of cooperation and competitive interactions, respectively. A community with a lower value of P or a higher relative fraction of |negative cohesion|: positive cohesion (N:P) indicated a more stable community. The vulnerability reflects how fast the consequence of microbial interactions affect either parts of or the entire network. Generally, a lower network vulnerability suggests a more stable community.

To identify potential interactions of the colonized gut microbiota in zebrafish, we constructed co-occurrence networks across zebrafish development as visualized by sampling points (only for those ≥ 27 replicates per time point, Fig. 2a). The networks generated at the OTU level indicated that the topology of all gut microbiota networks (except for 12 and 20 dph) fitted the power law distribution well (*R^2^* > 0.8), and therefore exhibited scale-free characteristics. Moreover, except for 70 dph, the modularity (the degree to which a network is compartmentalized into different modules) of each empirical network of gut microbiota was always significantly higher (*P < *0.05) than the corresponding randomized networks (Table S2), confirming the networks appear to be modular. However, the complexity of networks visualized by sampling points only significantly increased from 20 dph to 70 dph (Fig. 2b), and some key topological indices of the empirical networks, as visualized by sampling points, were not significantly different from the random networks (*P > *0.05, Table S2). We also found that only positive cohesion significantly decreased (*R^2^* = 0.64, *P = *0.03) with the sampling time. However, the relative fraction of |negative cohesion|: positive cohesion (N:P), which reflects the community stability, showed no significant relationship (*P > *0.05, Fig. 2c) with sampling time (i.e., dph). Also, the vulnerability, which reflects how fast the consequences of microbial interactions affect either part of or the entire network, showed no significant correlation (*P > *0.05, Fig. 2c) with sampling time.

Interestingly, the networks constructed for the different developmental stages (i.e., 12 to 26, 27 to 42, and 56 to 98 dph) indicated that the co-occurrence and interactions among networked OTUs in the last stage (56 to 98 dph) were more complex than those of the early stages (12 to 26 and 27 to 42 dph, Fig. S1a). Specifically, the total nodes and total links, as well as the average degree and connectedness tended to be much higher in the last stage (Fig. S1b). Some other key network topological indices such as geodesic efficiency (a higher value indicates that the nodes are closer), harmonic geodesic distance (a smaller value indicates all the nodes in the network are closer), and centralization of stress centrality (values close to 0 indicate a network where each node has the same stress centrality, and the value increases with greater difference among all stress centrality values) of the empirical networks were also significantly (*P < *0.05) different from those of the corresponding randomized stage-dependent networks (Table S2). These metrics indicated that such stage-dependent networks were non-random and unlikely due to chance. Therefore, to gain a better understanding of the assemblage patterns and network stability of zebrafish gut microbiota, the following results were mainly visualized according to the developmental stage rather than by sampling point.

### The networked communities differed significantly among zebrafish developmental stages.

**FIG 3:**
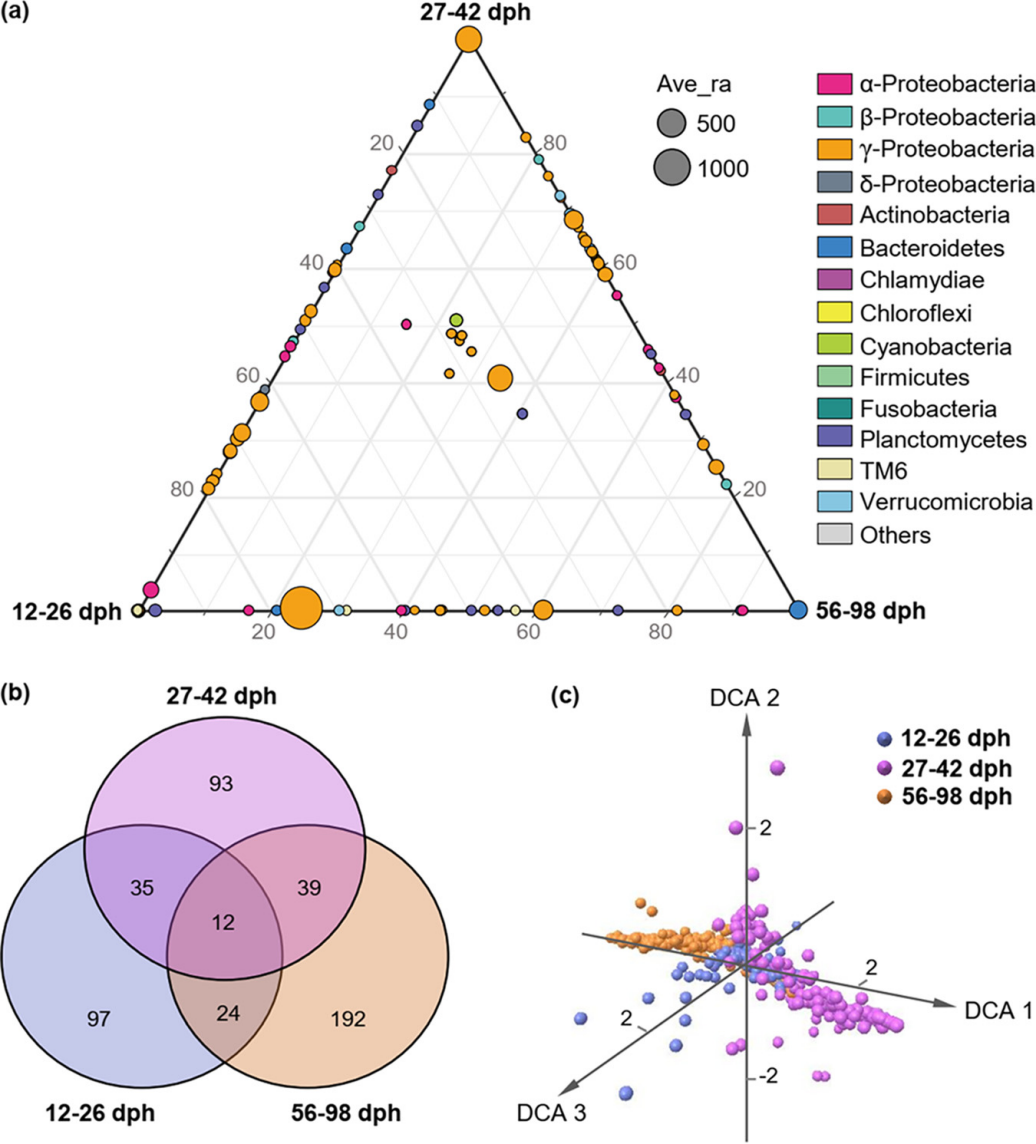
Distribution traits of the networked communities (assemblages of microbial taxa detected in the stage-dependent networks, and zebrafish development were divided into three stages as referred to in our previous study [5] according to the community patterns of gut microbiota). (a) Ternary plots of all networked OTUs (if an OTU was absent from a network, its abundance was set to 0 in all samples at that stage). Each circle represents an OTU, and its size represents the weighted average abundance. The position of each circle was determined by the contribution of the indicated compartments to the total relative abundance. (b) Venn diagrams showing the number of shared and unique network OTUs among developmental stages. (c) DCA ordination showing the dissimilarity of networked communities among developmental stages.

To specify the stage-dependent microbial networks across zebrafish development, assemblages of microbial taxa detected in the networks (i.e., networked communities) were further visualized by ternary plots (which effectively display compositional data and help to understand its multidimensional patterns), Venn plots, and detrended correspondence analysis (DCA) ordination. Ternary plots showed a clear selection of gut microbial taxa at different developmental stages. Specifically, the first two stages (12 to 26 and 27 to 42 dph) showed stronger selection for OTUs from the gammaproteobacteria, but there was stronger selection of Bacteroidetes (e.g., OTU16) in the last stage (56 to 98 dph). In addition, some taxa from alphaproteobacteria (e.g., OTU52) and Planctomycetes (OTU150) were also commonly selected at 12 to 26 dph (Fig. 3a). Venn plots indicated that there were many more unique networked OTUs at 56 to 98 dph (192) than at 12 to 26 dph (97) and 27 to 42 dph (93), whereas only 12 networked OTUs were common to all three stages (Fig. 3b). The common OTUs between 12 to 26 and 56 to 98 dph (24) were also fewer than any other two adjacent stages (35/39). DCA ordination showed that the structure of networked communities was considerably different among zebrafish developmental stages (Fig. 3c), which was further confirmed by the non-parametric dissimilarity analysis (MRPP, PERMANOVA, *P = *0.001, Table 1).

**TABLE 1:** Dissimilarity tests showing the dissimilarities of networked communities (assemblages of microbial taxa detected in the networks) across zebrafish development

Development	MRPP				PERMANOVA			
	Bray-Curtis		Jaccard		Bray-Curtis		Jaccard	
	*δ*	*p*	*δ*	*p*	*F*	*p*	*F*	*p*
According to sampling points^*a*^								
12 dph vs 20 dph	0.551	0.001	0.782	0.001	34.868	0.001	3.498	0.001
20 dph vs 27 dph	0.564	0.001	0.763	0.001	19.381	0.001	11.024	0.001
27 dph vs 42 dph	0.666	0.001	0.776	0.001	35.069	0.001	17.050	0.001
42 dph vs 56 dph	0.615	0.001	0.790	0.001	46.353	0.001	8.264	0.001
56 dph vs 70 dph	0.485	0.001	0.787	0.001	23.060	0.001	16.676	0.001
70 dph vs 98 dph	0.470	0.001	0.780	0.001	56.174	0.001	20.498	0.001
According to developmental stages^*b*^								
12 to 26 vs 27 to 42 dph	0.723	0.001	0.695	0.001	77.423	0.001	67.061	0.001
12 to 26 vs 56 to 98 dph	0.630	0.001	0.735	0.001	62.472	0.001	101.075	0.001
27 to 42 vs 56 to 98 dph	0.707	0.001	0.734	0.001	122.000	0.001	112.044	0.001

a Only for those with replicates ≥ 27 per time.

b The stages were divided refer to our previous study (5). MRPP, multi-response permutation procedure; PERMANOVA, permutational multivariate analysis of variance; dph, days post-hatching.

### Microbes from a previous developmental stage could be one of the major sources of gut microbiota for later stages.

SourceTracker analysis was performed to explore the deductive sources for assembling gut microbiota in zebrafish at different developmental stages. We found that microbial sources derived from an adjacent previous stage always accounted for the primary proportion, indicating a considerable part of the colonized gut microbes could be passed on to the next stage. Specifically, 78.36% of the gut microbial sources detected at 15 to 26 dph might be sourced from 12 dph; 67.29% of those at 27 to 42 dph sourced from 12 to 26 dph; and 79.5% of those at 56 to 98 dph sourced from 27 to 42 dph (Fig. S2a). Meanwhile, zebrafish gut microbes colonized from water environments only accounted for 0.04% to 0.05% in the early stages and increased to 2.32% in the last stage. The remaining 17.58% to 32.66% of microbial sources were classified as unknown (Fig. S2a). We also compared the affiliation of OTUs by exploring their sharedness or specificity between water microbiota and gut microbiota within developmental stages. We found that the number of OTUs only detected in water was always much smaller (5.46% to 15.58%) than that detected in the gut at the adjacent previous stage (22.64% to 39.23%, Fig. S2b). The shared OTUs in water and gut microbiota at the adjacent previous stage accounted for 27.92% to 52.76%, and the remaining 10.76% to 30.22% were classified as unknown (Fig. S2b).

### Modularity and connections of gut microbial networks increased with zebrafish development.

**FIG 4:**
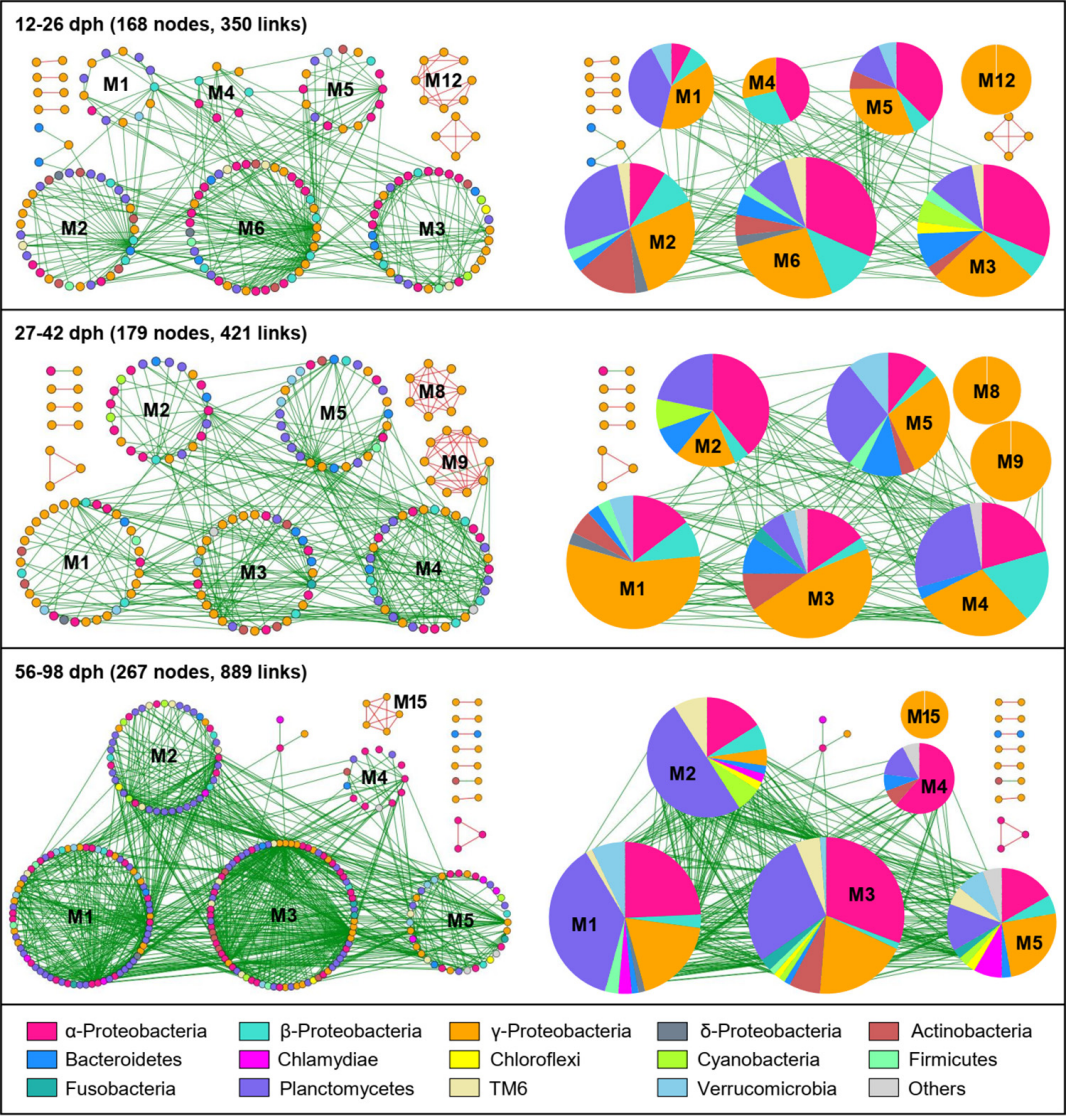
Network modules preserved across developmental stages. Large modules, with ≥ 5 nodes, are shown in circular layout for the constructed networks. Colors of nodes indicate major phyla (Proteobacteria further divided into classes). Red and green links indicate positive and negative correlations, respectively. The corresponding pie charts on the right panel for each stage-dependent network showing the proportions of major phyla (Proteobacteria further divided into classes). The module ID of each large module is indicated by M1 to M15.

The stage-dependent networks contained 12 to 15 modules with a modularity ranging from 0.374 to 0.489 (Table S2). Overall, taxa tended to co-exclude (negative correlations, green links) rather than co-occur (positive correlations, red links), with negative correlations accounting for 88.60 to 97.98% of observed links in the networks. To identify the possible variation of gut microbial interactions across zebrafish development, we focused on large modules (≥ 5 nodes) in the stage-dependent networks by highlighting the OTU correlations and the proportions of major phyla (Proteobacteria were further divided into classes). The network modules became larger and more connected as the zebrafish developed, as such the 56 to 98 dph network had the largest number of modules (15, Table S2) and the two largest modules (M1, M3) that included many more nodes (71, 78) than those in the early stages (i.e., 12 to 26 and 27 to 42 dph, Fig. 4). Moreover, the composition of OTUs in the modules also differed considerably across zebrafish development. Specifically, during the first stage (12 to 26 dph), gammaproteobacteria dominated the large modules (≥ 5 nodes), and primarily co-occurred with either alphaproteobacteria or Planctomycetes. At 27 to 42 dph, gammaproteobacteria increased considerably and became the major component of the large modules, especially in M1, M3, M8, and M9. However, the prominence of gammaproteobacteria at 56 to 98 dph decreased, while the primarily co-occurred alphaproteobacteria or Planctomycetes increased (Fig. 4).

### Potential keystone taxa and their effects on community patterns.

**FIG 5:**
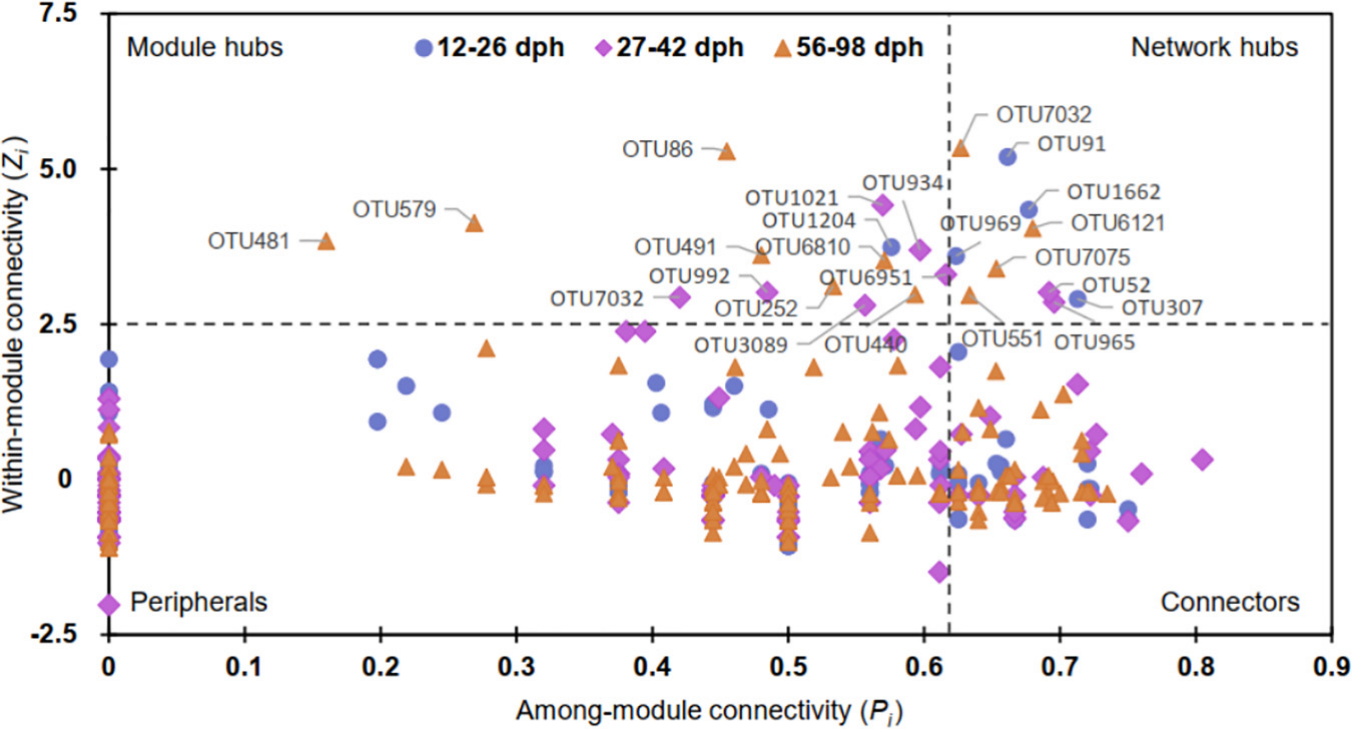
Classification of nodes to identify potential keystone OTUs within the stage-dependent gut microbial networks. *Z_i_* > 2.5 and *P_i_* > 0.62 indicates network hubs (highly connected nodes within entire network); *Z_i_* > 2.5 and *P_i_* ≤ 0.62 indicate module hubs (highly connected nodes within modules); *Z_i_* ≤ 2.5 and *P_i_* > 0.62 indicate connectors (nodes that connect modules); and *Z*_i_ ≤ 2.5 and *P_i_* ≤ 0.62 indicate peripherals (nodes connected in modules with few outside connections). The potential keystone taxa generally include network hubs, module hubs, and connectors.

The key network nodes, those with the maximal values of connectivity/betweenness (the ratio of paths that pass through the i^th^ node)/stress centrality (the number of geodesic paths that pass through the i^th^ node)/eigenvector centrality (the degree of a central node that it is connected to other central nodes), differed across zebrafish development. They were initially occupied by OTU1662 (*Serratia*) at 12 to 26 dph, then shared by OTU965 (*Vibrio*), OTU1021 (*Cetobacterium*), and OTU7097 (*Pseudoalteromonadaceae*) at 27 to 42 dph, but dominated by OTU7032 (*Pseudoalteromonadaceae*) at 56 to 98 dph. Most of these key network nodes were previously defined as “core microbiota” of the zebrafish intestine (1). The potential keystone taxa, those which significantly affect the networks and interactions of gut microbiota, were identified by the values of within-module connectivity (*Z_i_*) and among module connectivity (*P_i_*) of each OTU. Specifically, the potential keystone taxa included network hubs (*Z_i_* > 2.5 and *P_i_* > 0.62), module hubs (*Z_i_* > 2.5 and *P_i_* ≤ 0.62), and connectors (*Z_i_* ≤ 2.5 and *P_i_* > 0.62) (Fig. 5). We found that most of the identified potential keystone taxa (except for OTU141 at 12 to 26 dph) were not abundant in the gut microbiota (Table S3). At 12 to 26 dph, all identified network hubs (i.e., OTU91, OTU969, OTU307, OTU1662) and module hubs (i.e., OTU1204) were rare taxa (accounting for 0.004% to 0.019% of the abundance), as were most of the connectors (except for OTU141, OTU84, and OTU476). Similarly, most of the potential keystone taxa at 27 to 42 dph were rare taxa, with the exception of one network hub (OTU52, average 0.052%), one module hub (OTU1021, average 0.005%), and two connectors (OTU571 and OTU119, Table S3). In the last stage (56 to 98 dph), 75.00% of the identified network hubs, 42.86% of the module hubs, and 95.24% of the connectors were classified as rare taxa.

To verify which potential keystone taxa may affect the assemblage patterns of gut microbiota, the relationships between each potential keystone OTU and the alpha-diversity were examined. Results indicated that only the connectors showed significant correlations with all three diversity indices (i.e., phylogenetic distance [PD], richness, and Shannon), while none of the network hubs and module hubs did (Table 2). At 12 to 26 and 27 to 42 dph, individual sets of seven rare connectors were significantly (*P < *0.05) correlated with the alpha-diversity. However, there were 29 connectors that displayed significant (*P < *0.05) correlations with the alpha-diversity at 56 to 98 dph (Table 2). Such correlations were confirmed by the significant regression lines (*R*^2^ = 0.23-0.56, *P < *0.05) between the representative potential keystone OTUs (e.g., OTU335, OTU747, OTU677) and alpha-diversity indices (Fig. S3).

**TABLE 2:** The relationships between the alpha-diversity of gut microbiota and potential keystone OTUs

OTU iD	Avg abundance	Keystone type	Shannon		Richness		PD	
			*R*	*p*	*R*	*p*	*R*	*p*
12 to 26 dph	(%)							
OTU157	0.014	Connectors	0.525	<0.001	0.559	<0.001	0.476	<0.001
OTU204	0.022	Connectors	0.533	<0.001	0.000	0.001	0.426	0.001
OTU295	0.010	Connectors	0.409	0.006	0.603	<0.001	0.522	<0.001
OTU303	0.007	Connectors	0.538	<0.001	0.482	<0.001	0.470	<0.001
OTU335	0.007	Connectors	0.598	<0.001	0.658	<0.001	0.636	<0.001
OTU420	0.015	Connectors	0.542	<0.001	0.512	<0.001	0.483	<0.001
OTU1062	0.021	Connectors	0.507	<0.001	0.406	0.008	0.392	0.007
27 to 42 dph								
OTU436	0.017	Connectors	0.377	<0.001	0.327	0.007	0.324	0.004
OTU451	0.009	Connectors	0.381	<0.001	0.315	0.016	0.311	0.009
OTU465	0.015	Connectors	0.363	0.001	0.360	0.001	0.367	<0.001
OTU717	0.016	Connectors	0.403	<0.001	0.410	<0.001	0.383	<0.001
OTU747	0.006	Connectors	0.424	<0.001	0.349	0.002	0.345	0.001
OTU860	0.008	Connectors	0.356	0.001	0.327	0.007	0.301	0.016
OTU1625	0.004	Connectors	0.386	<0.001	0.340	0.003	0.290	0.029
56 to 98 dph								
OTU226^*a*^	0.052	Connectors	0.472	<0.001	0.512	<0.001	0.435	<0.001
OTU295	0.012	Connectors	0.367	<0.001	0.438	<0.001	0.000	<0.001
OTU296	0.024	Connectors	0.557	<0.001	0.644	<0.001	0.554	<0.001
OTU300	0.026	Connectors	0.561	<0.001	0.639	<0.001	0.556	<0.001
OTU322	0.024	Connectors	0.562	<0.001	0.653	<0.001	0.570	<0.001
OTU350	0.018	Connectors	0.378	<0.001	0.334	<0.001	0.334	<0.001
OTU354	0.019	Connectors	0.367	<0.001	0.287	0.002	0.279	0.002
OTU366	0.013	Connectors	0.609	<0.001	0.711	<0.001	0.675	<0.001
OTU390	0.014	Connectors	0.297	0.001	0.273	0.006	0.277	0.002
OTU424	0.015	Connectors	0.362	<0.001	0.313	<0.001	0.318	<0.001
OTU468	0.014	Connectors	0.356	<0.001	0.270	0.007	0.282	0.001
OTU482	0.008	Connectors	0.606	<0.001	0.693	<0.001	0.627	<0.001
OTU523	0.009	Connectors	0.497	<0.001	0.564	<0.001	0.490	<0.001
OTU526	0.007	Connectors	0.299	0.001	0.359	<0.001	0.377	<0.001
OTU542	0.010	Connectors	0.542	<0.001	0.602	<0.001	0.577	<0.001
OTU548	0.008	Connectors	0.538	<0.001	0.654	<0.001	0.576	<0.001
OTU578	0.010	Connectors	0.511	<0.001	0.536	<0.001	0.522	<0.001
OTU632	0.007	Connectors	0.570	<0.001	0.597	<0.001	0.558	<0.001
OTU677	0.007	Connectors	0.623	<0.001	0.651	<0.001	0.575	<0.001
OTU685	0.008	Connectors	0.583	<0.001	0.630	<0.001	0.564	<0.001
OTU687	0.006	Connectors	0.553	<0.001	0.637	<0.001	0.599	<0.001
OTU711	0.006	Connectors	0.506	<0.001	0.603	<0.001	0.556	<0.001
OTU857	0.004	Connectors	0.425	<0.001	0.488	<0.001	0.476	<0.001
OTU858	0.006	Connectors	0.266	0.010	0.333	<0.001	0.367	<0.001
OTU942	0.007	Connectors	0.368	<0.001	0.330	<0.001	0.303	<0.001
OTU947	0.005	Connectors	0.335	<0.001	0.357	<0.001	0.344	<0.001
OTU963	0.004	Connectors	0.329	<0.001	0.314	<0.001	0.281	0.001
OTU988	0.005	Connectors	0.427	<0.001	0.539	<0.001	0.506	<0.001
OTU6860	0.002	Connectors	0.381	<0.001	0.310	<0.001	0.273	0.002

a Indicates the OTU was neither abundant nor rare; no marks mean rare OTUs and their taxonomy (please see Table S3). Only the potential keystone OTUs showed significant (*p *< 0.05) relationships with all the three alpha-diversity indices were given. PD, phylogenetic distance; dph, days post-hatching.

### Zebrafish development increased network stability of gut microbiota.

**FIG 6:**
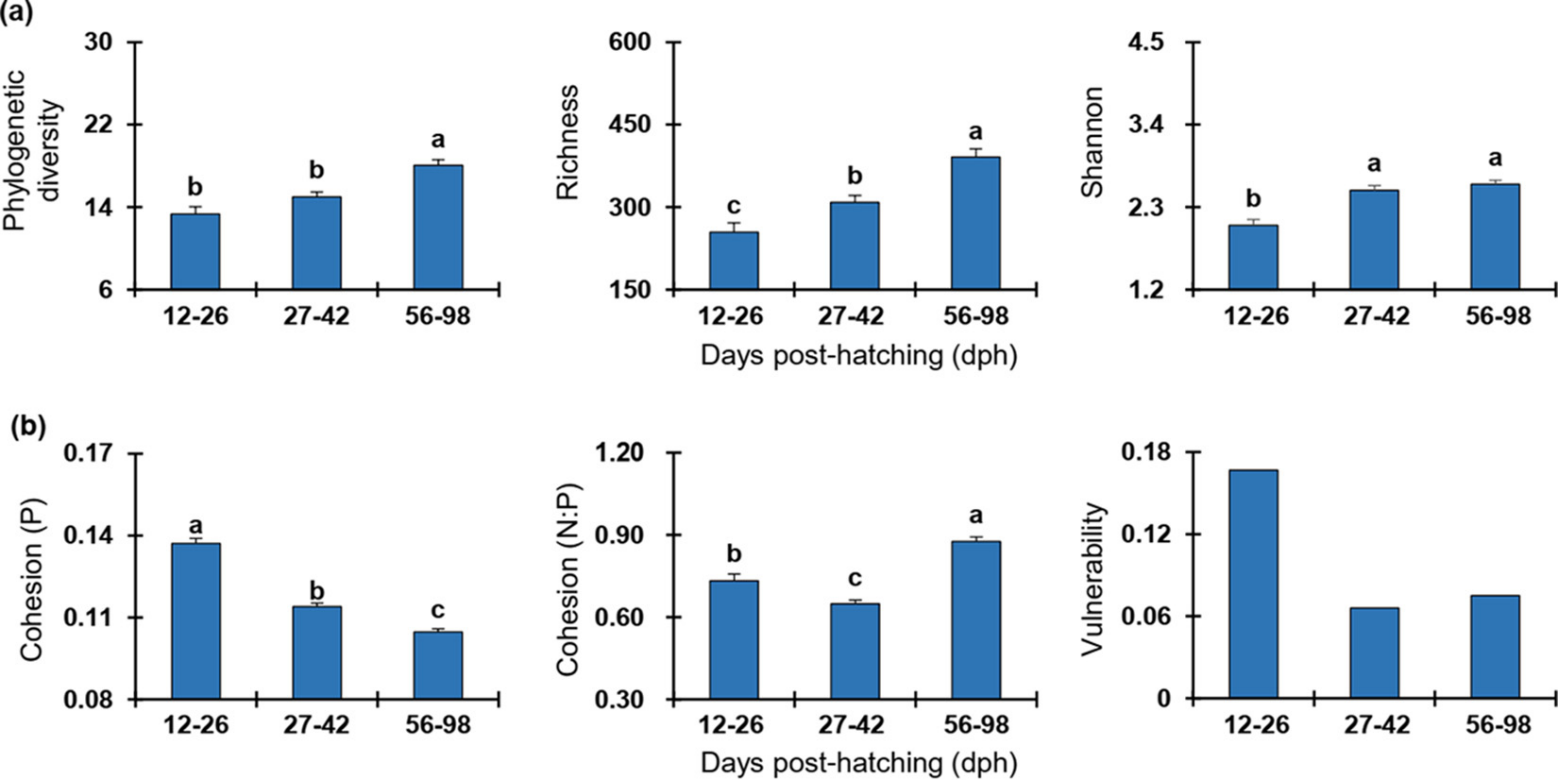
Variation of diversity and network stability of gut microbiota across zebrafish developmental stages. (a) Alpha diversity as visualized by zebrafish developmental stages. (b) Network stability as visualized by zebrafish developmental stages. Each error bar corresponds to the standard error. The variations among stages were tested through an ANOVA with least-significant-difference (LSD) tests. The presence of different letters denotes significant differences among stages, whereas the same letter indicates no statistical difference. However, the vulnerability of a network is indicated by the maximal vulnerability of nodes in the network. As there is no ANOVA test for the vulnerability, no letters are given for the vulnerability panel. The positive cohesion (P) and negative cohesion (N) reflect the magnitude of cooperation and competitive interactions, respectively. A community with a lower value of P or a higher relative fraction of |negative cohesion|: positive cohesion (N:P) indicates a more stable community. The vulnerability reflects how fast the consequence of microbial interactions affect either parts of or the entire network, and a lower network vulnerability suggests a more stable community.

The alpha- and beta-diversity indices all indicated that microbial community assembly in zebrafish varied significantly (*P < *0.05) across host developmental stages (Fig. 6a, Table S1). Specifically, the richness increased significantly (*P < *0.05) stage by stage, whereas the PD and Shannon of the last stage (56 to 98 dph) were significantly higher than those of the first stage (12 to 26 dph) (*P < *0.05). However, the diversity of the middle stage (27 to 42 dph) was similar to that of the first stage (e.g., PD) or the last stage (e.g., Shannon) (Fig. 6a). The dissimilarity tests based on the Bray-Curtis and Jaccard distances confirmed that the gut microbial diversity among stages were significantly different (MRPP, PERMANOVA, *P = *0.001) (Table S1). To understand whether and how zebrafish development affects the stability of gut microbiota, the stability-related indices (e.g., cohesion and vulnerability) were calculated from the empirical data. We found the positive cohesion significantly decreased (ANOVA, *P < *0.05) stage by stage, and the values of N:P for the last stage (56 to 98 dph) were also significantly higher than the early stages (12 to 26 and 27 to 42 dph, Fig. 6b) (ANOVA, *P < *0.05), indicating that gut microbial community in zebrafish becomes more stable in adults. Similarly, the network vulnerability in the first stage (12 to 26 dph) was much higher than that of the other stages (27 to 42 and 56 to 98 dph), confirming a more stable gut microbiota in adult zebrafish. In addition, the ratio of negative correlations among nodes was much higher in the last stage (56 to 98 dph, 97.98%) than in the early stages (92.86% and 88.60% at 12 to 26 and 27 to 42 dph, respectively), indicating a more stable network in adult zebrafish.

**FIG 7:**
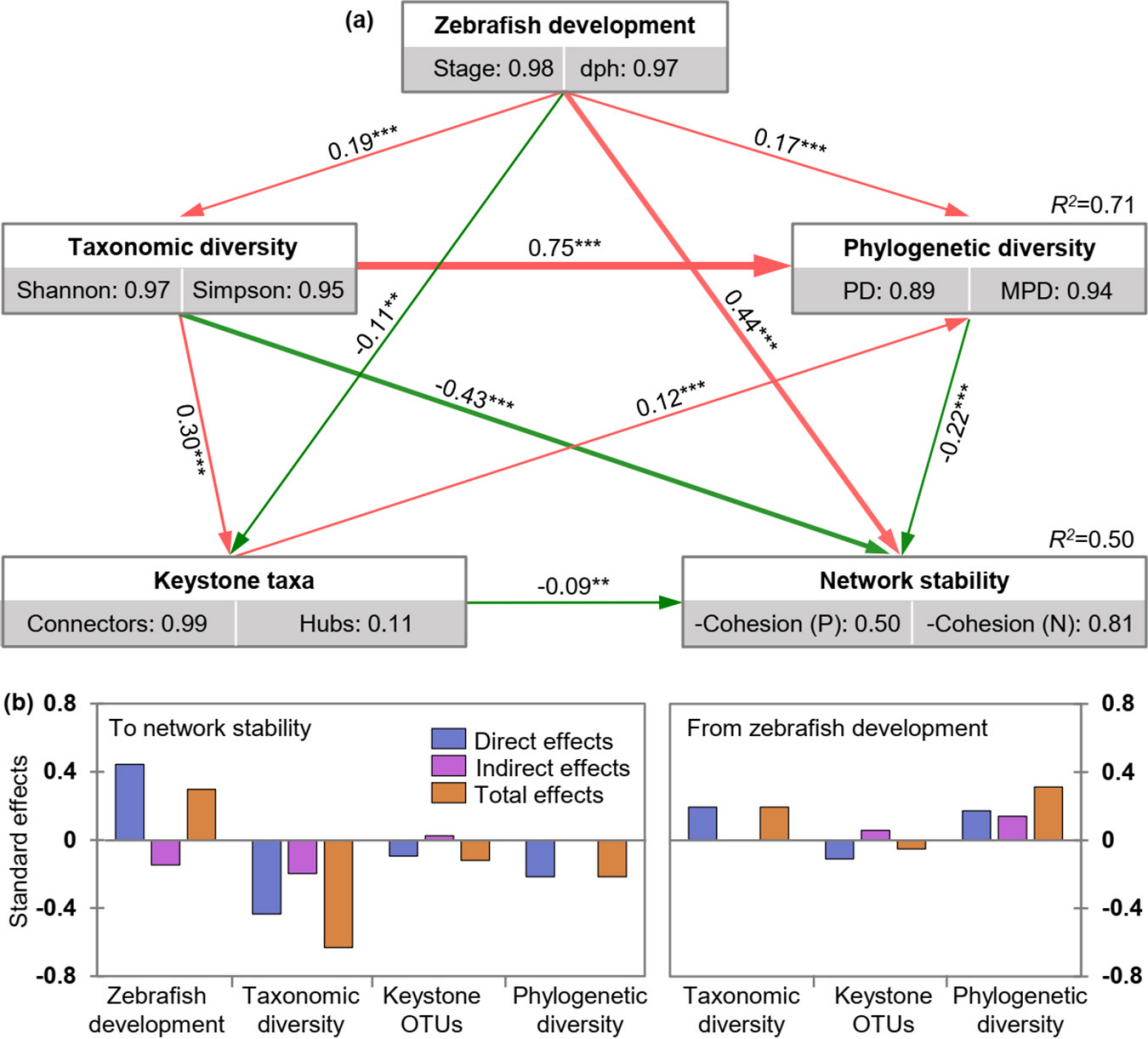
Effects of the major factors on the network stability as determined by the structural equation model (SEM) analysis. (a) Partial least-squares-path models showing the cascading relationships of different factors. Single-headed arrows indicate the hypothesized direction of causation. Rectangles represent the investigated components, and the numbers in the gray rectangles represent the positive relationship between manifest variables, which indicate that the manifest variables could reflect latent variables as well. Red and green solid lines with arrows indicate significant positive and negative relationships, respectively. The line width is proportional to the strength of the relationship. The numbers associated with arrows represent the direct effects of a latent variable to another latent variable. For example, the direct effect of zebrafish development to network stability is 0.44, and such values were calculated by constructing a reasonable data linear matrix using R packages of “plsmp.” The positive cohesion (P) and negative cohesion (N) reflects the magnitude of cooperation and competitive interactions, respectively. As the communities with lower values of P and N are more stable, their values were multiplied by −1 to make sure the stability retained the same trend with the variation of cohesion. (b) Standardized effects of different factors on the network stability or those from zebrafish development. The effect is called “standard effect” because its value was converted to range between −1 and 1. The direct effects were given by the path coefficients, while the indirect effects were obtained as the result of path coefficients by taking an indirect path. The total effects are the sum of both the direct and indirect effects. Asterisks indicate the statistical significance (*** *P < *0.001, and ** 0.001 < *P < *0.01); dph, days post-hatching; PD, phylogenetic distance; MPD, mean pairwise distance.

To quantify the effects of zebrafish development, microbial diversity, and potential keystone taxa on the stability of gut microbiota, structural equation modeling (SEM) was performed as illustrated by Fig. S4. The results indicated that zebrafish development was positively correlated (path coefficient = −0.44, *P < *0.001, Fig. 7a) with the stability, indicating network stability increased with zebrafish development. However, the taxonomic diversity, phylogenetic diversity, and keystone OTUs were negatively correlated (path coefficients = −0.43, −0.22, or −0.09, *P < *0.001 or 0.001 < *P < *0.01) with stability, indicating the network stability had a negative relationship with gut microbial diversity and potential keystone taxa. In addition, we also found that zebrafish development always showed significant (path coefficients = 0.19, 0.17 or −0.11, *P < *0.001 or 0.001 < *P < *0.01) effects on the gut microbial diversity and potential keystone taxa (Fig. 7).

## DISCUSSION

Understanding gut microbial interactions and stability is an important but largely ignored ecological issue, which will be able to guide gut microbial management for providing better ecological services (29). By analyzing the molecular ecological networks of gut microbiota from more than 550 zebrafish, we found that zebrafish development significantly increased microbial interactions in the gut ecosystem, and the microbial ecological networks in adult zebrafish were more stable than that of larval fish. This finding supports our core hypothesis that gut microbial stability would increase with fish development due to enhanced microbial interactions. Thus, consideration of fish developmental stage in maintaining the stability of fish gut ecosystem through microbial management may be a priority, with the adult stage seeming to be a more reasonable intervention time than that of the larval stage. Although some current studies have realized that 4 or 5 years of gut microbiome stability contributes considerably to human health (22, 23), such temporal scale is still far from enough to address the microbial succession over the entire life cycle of a human. However, by analyzing human fecal samples from individuals aged from 0 to 70 years, Yatsunenko et al. (30) found that gut microbial diversity differed considerably from children to adults. Thus, regardless of whether lower (e.g., fish) or higher (e.g., human) vertebrate, it is necessary to examine numerous individuals of different ages to obtain the full profile of gut microbiota.

By analyzing zebrafish ranged from 12 to 98 dph, we found that microbial diversity, interactions, and stability of fish gut microbiota increased with host development, and that the gut microbial patterns could be divided into three stages. The findings obtained with this larger data set (> 550 fish individuals) confirmed our previous study that found fish development was one of the most important forces driving the ecological succession of gut microbiota. The variations of host immunity and gut nutrient niches across fish development could significantly affect the gut microbiota (5). In zebrafish, immunity, as profiled by the expression of immune-related genes, suggested that it generally matures by 4 to 6 weeks post-fertilization (31). Development of the immune system in zebrafish could increase the ecological selection on microorganisms in the gut ecosystem. Specifically, adaptive immunity in zebrafish in the form of B- and T-cell responses is fully formed by 4 weeks post-fertilization and the adaptive immunity helps to drive microbial composition within zebrafish (6, 32). Therefore, the adaptive immunity might increase the gut microbial network stability in adult zebrafish over that of larval fish. Also, the space available for colonizing microorganisms in the gut ecosystem increases with zebrafish developing days, which could be visualized by the increase of intestinal volume across zebrafish development (27). Generally, a large area/volume of ecosystem can house more species (33) due to the species–area/volume relationship power law (27, 34). Thus, our results herein indicated that the gut microbial diversity at the adult stage was significantly higher than that of larval zebrafish, which was also consistent with that of humans (30). High diversity generally enhances the ecosystem stability, and mounting evidence supports the positive diversity-stability relationship (35). On the other hand, the available nutrients in the gut ecosystem continuously vary across fish development, and the nutrient niche theory suggests that the nutrients determine which microorganisms can successfully colonize in the gut (36). The fixed-food supply and relatively stable metabolic activities of the adult stage might decrease the heterogeneity of nutrient niche, which is likely critical for ecosystem stability (37). Such development-dependent characteristics in zebrafish therefore collectively contribute to maintain a more stable gut microbiota in zebrafish at the adult stage than larval stage.

We also found that the zebrafish gut microbial network of the last developmental stage showed a higher proportion of negative correlations, indicating higher ecosystem stability than that of the first two stages. Generally, cooperating networks of microbes tend to be unstable, but microbial competition could enhance the stability (16), that means stability could be promoted by increasing negative correlations or limiting positive feedbacks. Moreover, previous research has indicated that keystone taxa in a community contribute significantly to the maintenance of ecosystem stability (38) due to their strong effects on the network structure and interactions (39). The removal of keystone taxa may result in disassemble modules or networks, and thus tends to weaken the ecosystem stability (40). Previously discovered “core microbiota” (e.g., *Vibrio,* Pseudomonas, *Cetobacterium*) in zebrafish intestines (1) were identified as potential keystone taxa in the present study and might act as key network nodes to maintain zebrafish gut ecosystem stability. Recent studies have also suggested that keystone taxa might be indispensable for microbiome recovery in the human gut ecosystem after disturbances (41, 42), confirming the importance of keystone taxa in affecting stability. In addition, our results indicated there were many more potential keystone taxa associated with the diversity of gut microbiota during the last developmental stage, suggesting that gut microbial stability is enhanced in adult zebrafish.

However, the potential keystone taxa with disproportionately large effects on gut microbial stability in zebrafish showed relatively low abundances and were generally classified as rare taxa. Recently, there has been increasing evidence indicating that rare taxa in ecosystems have important ecological roles such as serving as reservoirs of genetic and functional diversity (43). Under the appropriate conditions, they can increase their abundance quickly to play particular functions and maintain ecosystem stability. For example, beneficial bacteria such as those of the family Pseudoalteromonadaceae (gammaproteobacteria) increase in abundance in the intestine after dietary replacement of fishmeal with soybean protein concentrate, which may help the host by enhancing disease resistance (44). This may help explain why rare Pseudoalteromonadaceae members, such as OTU6121, OTU7032, and OTU7075, were identified as potential keystone taxa to maintain the gut microbial network at the adult stage: they may remain at a low abundance under specific dietary conditions, but could increase due to dietary changes at the adult stage, maintaining ecosystem stability. In contrast, intestinal microbiota dysbiosis in the intestine exemplifies the “microecological Koch’s postulates” from an ecological perspective (45). Fortunately, rare microorganisms are able to be detected with the help of high-throughput methods (46), and increasing evidence has indicated that rare members also could serve as keystone taxa due to their outsized effects on the ecosystem (47). Additionally, the increase of Planctomycetes members in identified modules from the last stage was consistent with previous nutritional programming study in zebrafish, which found Planctomycetes displayed higher abundance in zebrafish gut at 65 dph compared with the early stages (6 to 36 dph) (48). However, we should acknowledge that the exact roles played by potential keystone taxa and the underlying mechanisms still need further validation by germfree zebrafish studies.

In summary, by examining the gut microbial network dynamics and stability in a zebrafish model across an entire life cycle, we found that microbial interactions and stability in the gut ecosystem increase with zebrafish development. These findings have several important implications for future microbial management to provide better gut ecosystem services for the host. First, like the clear diversity patterns of gut microbiota found across the fish development, microbial interactions and stability in the fish gut ecosystem were also closely correlated with host development. Thus, future studies should consider utilizing different fish age groups to gain a better understanding of gut microbial networks. Second, fish development also significantly affects the potential keystone taxa (rare but not necessarily abundant) of gut microbiota and result in high stability at the adult stage. Therefore, gut microbial management of the rare keystone taxa at the adult stage may be a good way to regulate gut ecosystem stability. Further clarification of how to precisely regulate the promising targets of rare but key taxa will be useful for maintaining beneficial host-associated microbial communities and controlling microbes-associated diseases (49).

## MATERIALS AND METHODS

### Zebrafish (Danio rerio) husbandry and experimental design.

This study mainly aimed to specify the microbial interactions and gut ecosystem stability across zebrafish development after hatching from the embryo (generally 3 days post-fertilization). Therefore, we used days post-hatching (dph) to record the sampling times. The same batch of fertilized zebrafish (AB strain) embryos obtained from China Zebrafish Resource Center were hatched using water from three different environments (5). Then, the hatched zebrafish in each environment were transferred to three independent tanks (130 × 30 × 40 cm) at 12 dph, but raised in small net cages fixed in the tanks as described previously (5). In each tank, there were three cages to separate fish transferred from environments A, B, and C, respectively. However, the cages within each tank were connected and shared the same water. Zebrafish were reared using a stable water temperature (28 ± 0.5°C) and a 14/10 h light/dark cycle was applied throughout the experiment. No additional food was given before 4 dph as the yolk sac was not completely consumed. Then, zebrafish were fed twice daily (9:00 and 15:00, respectively) with cultured *Paramecium* (5 to 8 dph), 20 μm mesh filtrated boiled egg yolk (9 to 11 dph), live brine shrimp (12 to 19 dph), and a standard dry fish food from 20 dph onward. To decrease the possible effects of diets, which changed more frequently before 12 dph, we chose the 12 dph as the first sampling point to analyze zebrafish gut microbiota. During the experiment, fully aerated tap water was used without filtering, and no recirculation occurred among the three tanks to make each tank independent. The zebrafish gut samples analyzed herein, included the 189 samples published previously (5) with an additional 364 samples, were nearly triple of our previous study (5), and this study used a much more intensive sampling regime (19 times, Fig. 1a). The sampling intervals were mostly 1 week (Fig. 1a); however, 1 to 14 days intervals were also applied occasionally to address gut microbial variations within different days across zebrafish development. At each sampling time, we randomly selected at least three zebrafish individuals from each tank (i.e., 9 replicates from 3 tanks). However, to visualize the interactions and stability of gut microbiota by ecological network analysis, we increased the replicates from 27 to 90 at the sampling points of 12, 20, 27, 42, 56, 70, and 98 dph (Fig. 1a). Many more but different replicates were applied for these sampling points to decrease the possible sample effects involved in the network analysis. The intestines of larval individuals (12 to 30 dph) were immediately removed aseptically under a dissecting microscope as described previously (27), while the juvenile/adult individuals had their intestines directly aseptically removed. The whole intestine of each fish was collected as a single sample, ensuring the gut microbial diversity would not be underestimated due to insufficient sampling. To test the possible sources of gut microbes that may colonize from the surrounding water, and for comparing the gut microbiota and water microbiota, we also collected water samples from the left (L), center (C), and right (R) of each tank from 15 to 98 dph. For each water sample, 500 mL of water was sequentially filtered through 1.2-mm (Whatman, NJ, USA) and 0.22-mm filters (Millipore, MA USA) to collect microbial cells in the water (50). The collected fish intestines and filters were immediately stored at −80°C until DNA extraction.

All protocols involved in the zebrafish experiments were approved by the Institutional Animal Care and Use Committee of the Institute of Hydrobiology, Chinese Academy of Sciences (Approval ID: Keshuizhuan 08529).

### DNA extraction and 16S rRNA gene sequencing analysis.

Genomic DNA of each collected sample was extracted using the PowerFecal (gut samples) or PowerWater (water samples) DNA isolation kit (Mo Bio, CA, USA) following the manufacturer’s instructions. The concentrations and quality of extracted DNA were determined using a NanoDrop One spectrophotometer (Thermo Fisher Scientific, MA, USA). Individual samples for which DNA extraction failed were excluded from the following analysis. In total, 652 genomic DNAs (553 and 99 from gut and water samples, respectively) were obtained. All the DNA samples were then diluted to 10 ng/μL for subsequent PCR amplification. The V4 to V5 regions of the 16S rRNA gene was amplified by the primer set of 515F (5′-GTGCCAGCMGCCGCGGTAA-3′) and 907R (5′-CCGTCAATTCMTTTRAGTTT-3′). Each sample was amplified in a 50-μL reaction system containing 1x Premix *Taq* DNA polymerase (buffer, dNTP, and *Taq* were included), 0.2 mM each primer, and 50 ng genomic DNA using the following program: DNA pre-denaturation for 5 min at 95°C; then 30 cycles of 30 s at 95°C, 30 s at 52°C, and 30 s at 72°C; followed by a post-amplification extension of 10 min at 72°C. PCR products were visualized using 1% agarose gels stained with ethidium bromide, and negative controls were always performed to ensure that no contamination had occurred.

After all samples were successfully amplified, DNA quantification was performed using a PicoGreen dsDNA assay kit (Invitrogen, CA, USA) according to the manufacturer’s instructions. To ensure an identical sequencing depth for each sample, PCR products were equally combined and mixed fully. All mixtures were added to a 2% agarose gel and the excised target DNA band was purified with a QIAquick Gel Extraction Kit (Qiagen, CA, USA). After a re-quantification of the purified DNA, a library was constructed using a NEBNext Ultra DNA Library Prep Kit (Biolabs, MA, New England) following the manufacturer’s instructions. The constructed library was sequenced on the Illumina HiSeq 2500 platform (Illumina, CA, USA) by Guangdong Magigene Biotechnology Co., Ltd. using a 2 × 250 bp kit (Illumina, CA, USA).

Quality filtering and processing of sequencing reads were conducted according to the methods as described previously (5) using a publicly available Galaxy pipeline (http://mem.rcees.ac.cn:8080/) (51). The overlapped paired-end sequences were first assembled using quantitative insights into microbial ecology (QIIME) (52), and poorly overlapped and low-quality sequences such as those with length <140 bp and a moving-window (5 bp) quality score < 20 were excluded before downstream analysis. The zOTUs (hereinafter referred to as OTU) were generated by the UNOISE method, and all samples was rarefied to the same sequencing depth (14,666 sequences per sample) prior to subsequent analysis.

### Microbial sources analysis.

SourceTracker analysis, a Bayesian approach to estimate the proportion of microbes in a given community that come from possible source environments (53), was used to identify possible microbial sources and estimate their relative contribution as proportions to the assemblage of gut microbiota (i.e., the sinks). As zebrafish gut microbiota showed clear assemblage patterns according to zebrafish development (5), the SourceTracker analysis were performed stage by stage (i.e., 12 to 26, 27 to 42, and 56 to 98 dph) to specify the sinks at different stages. Our SourceTracker models included water microorganisms and gut microbes from the adjacent previous stage as sources. To better exhibit the source contribution from the adjacent previous stage, we used the gut microbes colonized at 12 dph (i.e., the first sampling point) as sources for the assembly of gut microbiota at 15 to 26 dph. Independent SourceTracker analysis was carried out for each sink, but only the mean proportion of every source were given at each stage. In addition, the detected OTUs within stages were also directly compared by determining their affiliation (i.e., sharedness or specificity) with water microbiota or gut microbiota of the adjacent previous stage.

### Microbial network construction.

To explore microbial interactions and network stability of gut microbiota across zebrafish development, network analysis was performed using the Molecular Ecological Network Analysis (MENA) pipeline (http://ieg2.ou.edu/MENA/) as described previously (54). Our previous study (5) with a small data set (189 samples) as well as the larger data set herein (553 gut samples) indicated that both hatching and husbandry environments had little impact on zebrafish gut microbial patterns. Thus, networks were constructed for gut microbial communities based on OTU relative abundances at each sampling point or developmental stage regardless of the environments. Covariations were measured across 27 to 90 (time point-dependent networks) or 108 to 274 (stage-dependent networks) biological replicates to create each network. Only OTUs detected in 20% of replicate samples were retained for network construction. The Spearman coefficient was calculated based on the log-transformed relative abundances of each OTU. Random matrix theory (RMT) was used to automatically identify the appropriate similarity threshold (*St*) prior to network construction (55). Then, all gut microbial networks were constructed using the same *St* (i.e., 0.802). To ensure the reliability, networks were only constructed for sampling points with more than 27 replicates per time point (including 12, 20, 27, 42, 56, 70, 98 dph), whereas those with only 9 replicates were not involved in the time point-dependent network construction. The molecular ecological networks (MENs) were also constructed according to host developmental stage, which were divided into the three stages as referred to in our previous study (i.e., 12 to 26, 27 to 42, and 56 to 98 dph) (5). In addition, a network including all 99 water samples was also constructed using the same *St* (i.e., 0.802). All constructed networks were visualized using Gephi 0.9.2 and Cytoscape 3.8.2.

### Network characterization.

Global network properties were characterized according to Deng et al. (54). Briefly, to characterize the topological structure of the constructed MENs, various network topological indices such as number of nodes and links, average degree and connectedness were calculated. The correlation coefficients between pairs of nodes within each network were used as the weights of links. The significance of the constructed empirical MENs was tested by generating 100 random networks for each empirical network. During randomization, the same suite of network topological properties was calculated as that of the constructed empirical MENs. The means and standard deviations of each property calculated from the 100 randomizations were compared with the corresponding empirical MENs (15, 54).

To screen potential keystone taxa that may affect the assemblage patterns of gut microbiota, the connectivity of each node (i.e., OTU) in networks was evaluated by its within-module connectivity (*Z_i_*) and among-module connectivity (*P_i_*), which were calculated as follows:

$$Z_{i}=\frac{k_{\mathrm{ib}}-{k¯}_{b}}{\sigma_{\mathrm{kb}}}$$

$$P_{i}=1-\overset{N_{M}}{\underset{c=1}{\Sigma }}(\frac{k_{\mathrm{ic}}}{k_{i}})2$$

where k*_ib_* is the number of links of node *i* to all other nodes in module *b*; ${\bar{k}}_{b}$ and *σ_kb_* are the mean and standard deviation of within-module connectivity, respectively; *N_M_* is the number of modules in the whole network; *k_i_* is the number of links of node *i* in the whole network; *k_ic_* is the number of links from node *i* to all other nodes in module *c*. This can classify all nodes into four categories: *Z_i_* > 2.5 and *P_i_* > 0.62 indicated network hubs (highly connected nodes within entire network); *Z_i_* > 2.5 and *P_i_* ≤ 0.62 indicated module hubs (highly connected nodes within modules); *Z_i_* ≤ 2.5 and *P_i_* > 0.62 indicated connectors (nodes that connect modules); and *Z*_i_ ≤ 2.5 and *P_i_* ≤ 0.62 indicated peripherals (nodes connected in modules with few outside connections) (54). In general, the network hubs, module hubs, and connectors can be regarded as potential keystone taxa (40, 54), and these were further classified as abundant or rare taxa following the guidelines of recent studies (56, 57). Briefly, OTUs in a regional community (i.e., within stages) that had a mean relative abundance of > 0.1% were classified as abundant taxa, which also include locally abundant OTUs with a relative abundance ≥ 0.01% in all samples and ≥ 1% in some samples. Rare OTUs were those with a local abundance < 0.01% in some samples but never ≥ 1% in any sample. The remaining OTUs with a relative abundance generally < 0.01% but ≥ 1% in some samples are classified as neither rare nor abundant taxa (56, 57).

### Network stability analysis and structural equation modeling analysis.

The indices of cohesion and vulnerability were used to evaluate the microbial network complexity and stability across zebrafish development. The cohesion was calculated for each community to quantify the microbial connectivity according to the protocol of Herren and McMahon (58). Positive cohesion (P) reflects the magnitude of cooperation, which may reduce community stability (16), while negative cohesion (N) indicates the degree of competitive interactions among OTUs in a community. Communities with lower values of P and N are more stable. The relative fraction of |negative cohesion|: positive cohesion (N:P) was also used as one of the properties to reflect community stability (59), where a higher value of N:P indicated a more stable community. Thus, to ensure that stability displayed the same trend as the variation of cohesion, the values of P and N were multiplied by −1 in the structural equation model (SEM) analysis to determine which factors significantly affected the stability. Also, we calculated the vulnerability using the method and R codes as described previously by Yuan et al. (15). The vulnerability of each node measures the relative contribution of the node to the global efficiency. The vulnerability of a network, which is indicated by the maximal vulnerability of nodes in the network, was then used to reflect how fast the consequence of microbial interactions affect either parts of or the entire network. Generally, a lower network vulnerability suggests a more stable community. In addition, the network characteristics such as the numbers of nodes and links, which could correlate with interactions and stability, were also visualized by the analysis of variance (ANOVA, among the three developmental stages) or regression analysis (among sampling points) across zebrafish development. The ratio of negative correlations to total links among nodes in each network was also used for reflecting the stability, with a higher negative correlation ratio generally indicating a more stable network (16).

The SEM was performed using R package of “plsmp” (60) to quantify the effects of different factors (i.e., zebrafish development reflected by stage and dph; taxonomic diversity reflected by Shannon and Simpson; phylogenetic diversity reflected by PD and mean pairwise distance [MDP]; keystone taxa box was reflected by connectors and hubs) on the stability of gut microbiota, which indicated by positive (P) and negative cohesion (N). We first constructed *a priori* model with latent variables, manifest variables, and path diagram (Fig. S4). Latent variables were hypothetical variables that could not be measured directly and were taken as underlying variables that help explain the association between two or more manifest variables.

### Statistical analysis.

The zOTUs of gut microbiota and networked communities (assemblages of microbial taxa detected in the networks), as well as parameters calculated accordingly involved one individuals or all of the following statistical analyses: (i) alpha-diversity comparisons were conducted to reveal dynamics of gut microbiota throughout zebrafish development as visualized by sampling points and developmental stages; (ii) significance tests were performed through an ANOVA with least-significant-difference (LSD) to examine whether differences among comparisons were significant or not; (iii) correlation analysis was performed to ascertain whether the characteristics interested were significantly correlated or not (61); (iv) nonparametric dissimilarity tests including multiple-response permutation procedure (MRPP) and permutational multivariate analysis of variance (PERMANOVA) tests were performed to compare community dissimilarities based on Bray-Curtis and Jaccard distances, respectively (62); (v) ternary plots, Venn plots, and DCA were used to compare the networked communities among stages as with our previous study (63). All the statistical analyses were performed based on the R software (R Foundation for Statistical Computing, Vienna, Austria) if not specified otherwise.

### Data availability.

The 16S rRNA gene sequencing data are available in the National Omics Data Encyclopedia (NODE) database with an accession number OEP002082.
