# Supplementary material for: Interactions and Stability of Gut Microbiota in Zebrafish Increase with Host Development
Source: Microbiol Spectr. 2022 Mar 21;10(2):e01696-21. doi: 10.1128/spectrum.01696-21 (PMC9045336; doi:10.1128/spectrum.01696-21)
Supplement: SUPPLEMENTAL FILE 1 — Supplemental material. Download SPECTRUM01696-21_Supp_1_seq11.pdf, PDF file, 0.7 MB [file spectrum01696-21_supp_1_seq11.pdf]

Supplemental Material

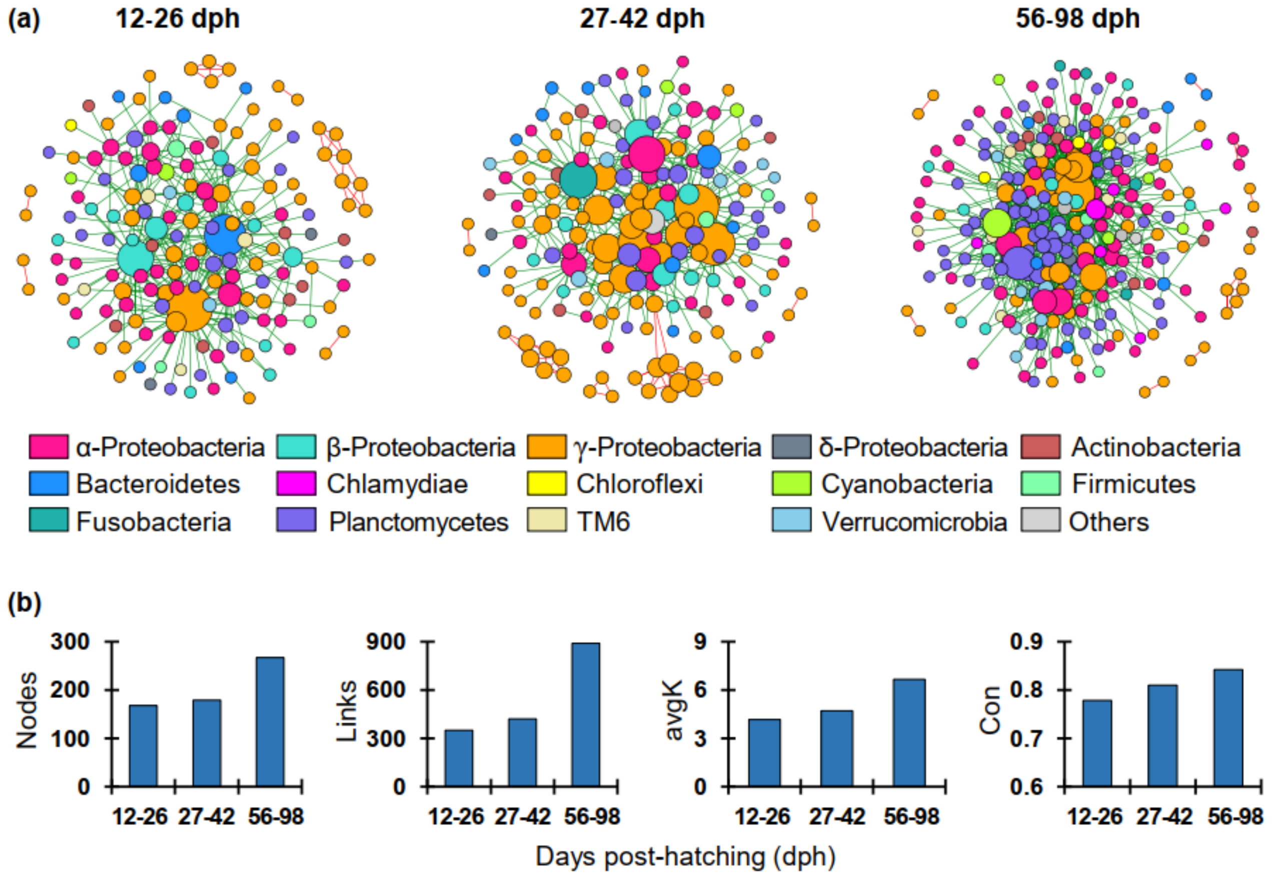

Fig S1 Succession of gut microbial networks over zebrafish developmental stages. (a) Visualization of the constructed stage-dependent networks showing the node interactions with emphasize on the taxonomy and node degree of different OTUs. Each node represents an OTU and the node degree as indicated by the size represents the strength of connections among OTUs, and colors of nodes indicate phyla (Proteobacteria further divided into class). Red and green links indicate positive and negative correlations, respectively. Details of network topological attributes are summarized in Table S2. (b) Stage-dependent changes of network topology including nodes, links, average degree (avgK), and the connectedness (Con).

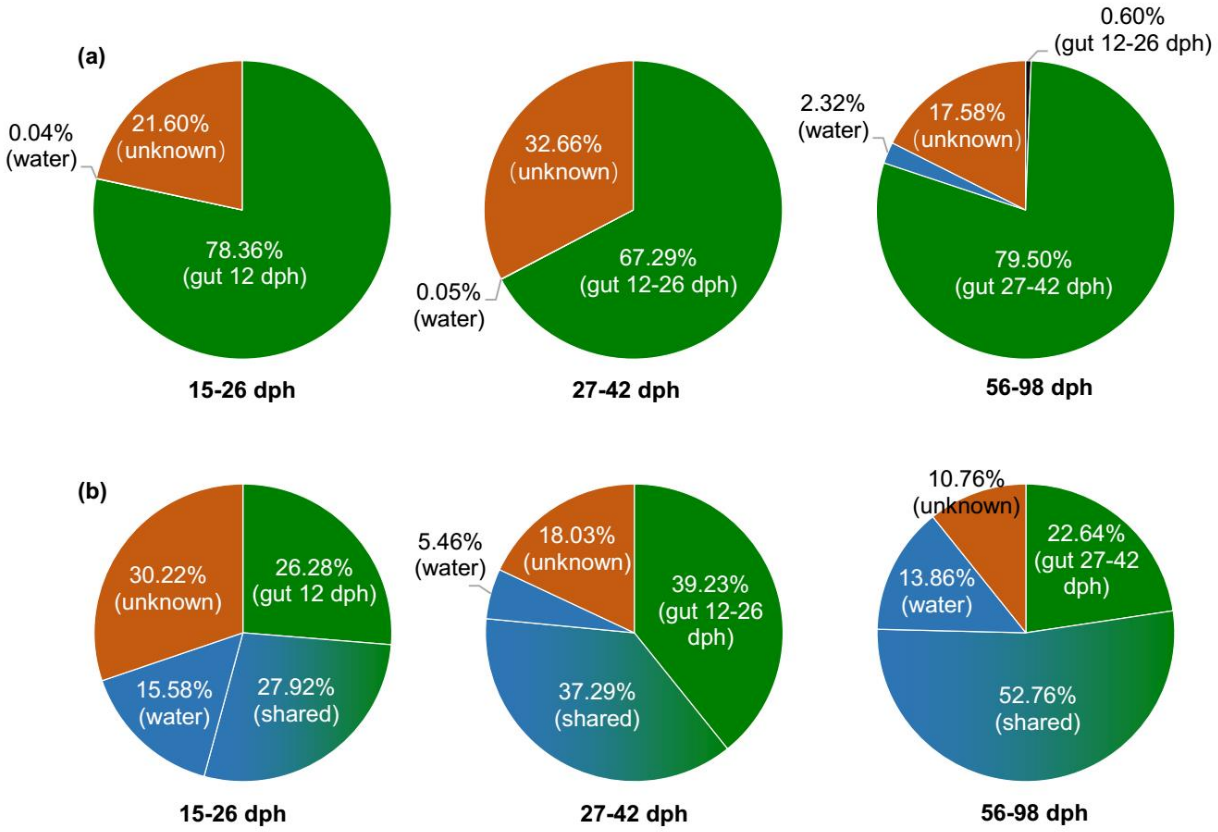

Fig S2 Microbial sources of zebrafish gut microbiota. (a) SourceTracker proportion estimates for a subset of sink at each developmental stage. Water microbiota and gut microbiota of the adjacent previous stage were used as sources. (b) Percentage of OTUs that come from different sources. The shared (indicated by mixed colours) means the OTUs are shared by water and gut microbiota.

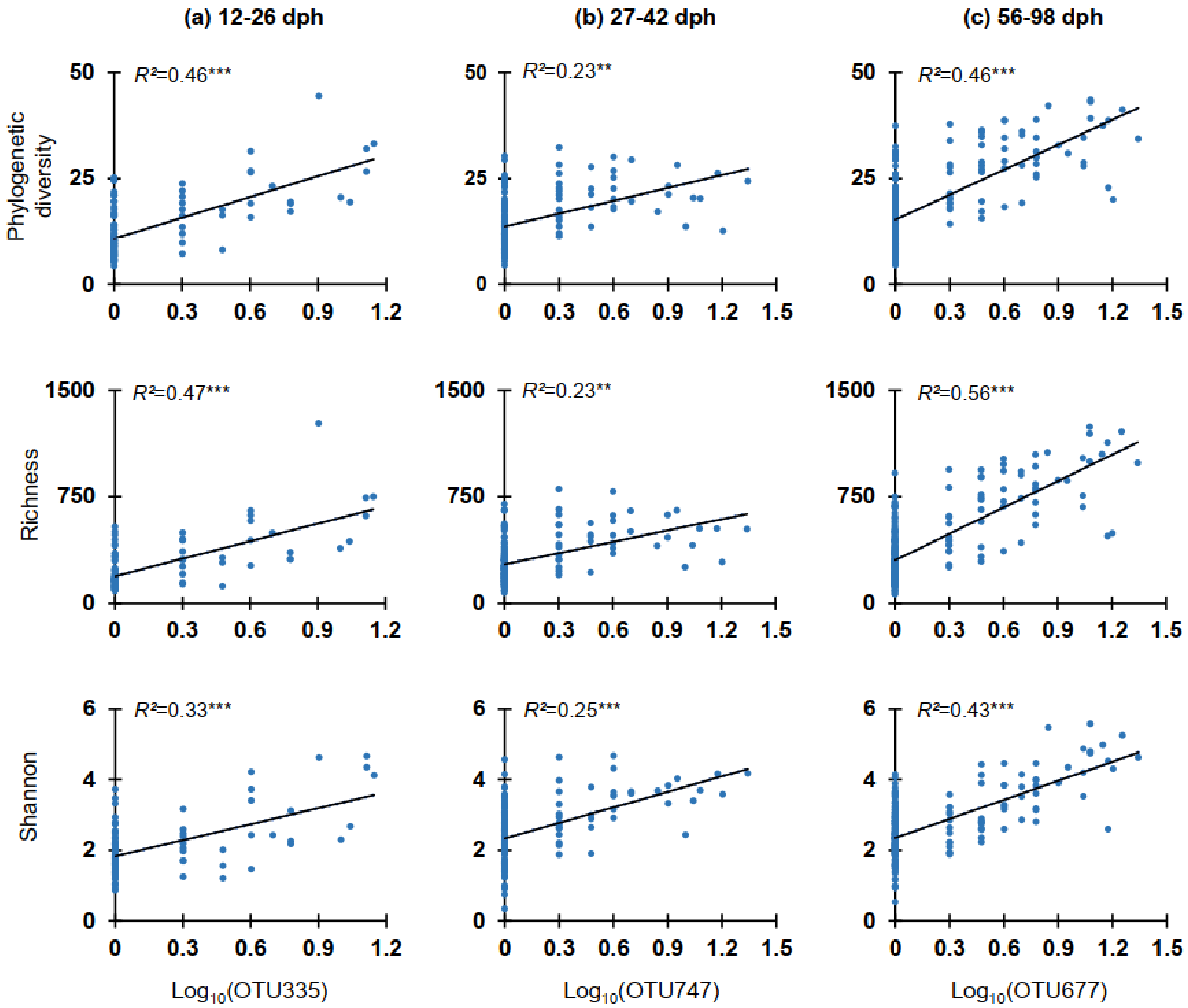

Fig S3 Alpha-diversity of gut microbiota significantly increased as the increasing of keystone OTU's abundance. Only given a representative OTU for each developmental stage, and the asterisks indicate the statistical significance (*** $p < 0.001$, and ** $0.001 < p < 0.01$). OTU335: *Rhizobiales*; OTU747: *Legionellaceae*; OTU677: *Planctomyces*.

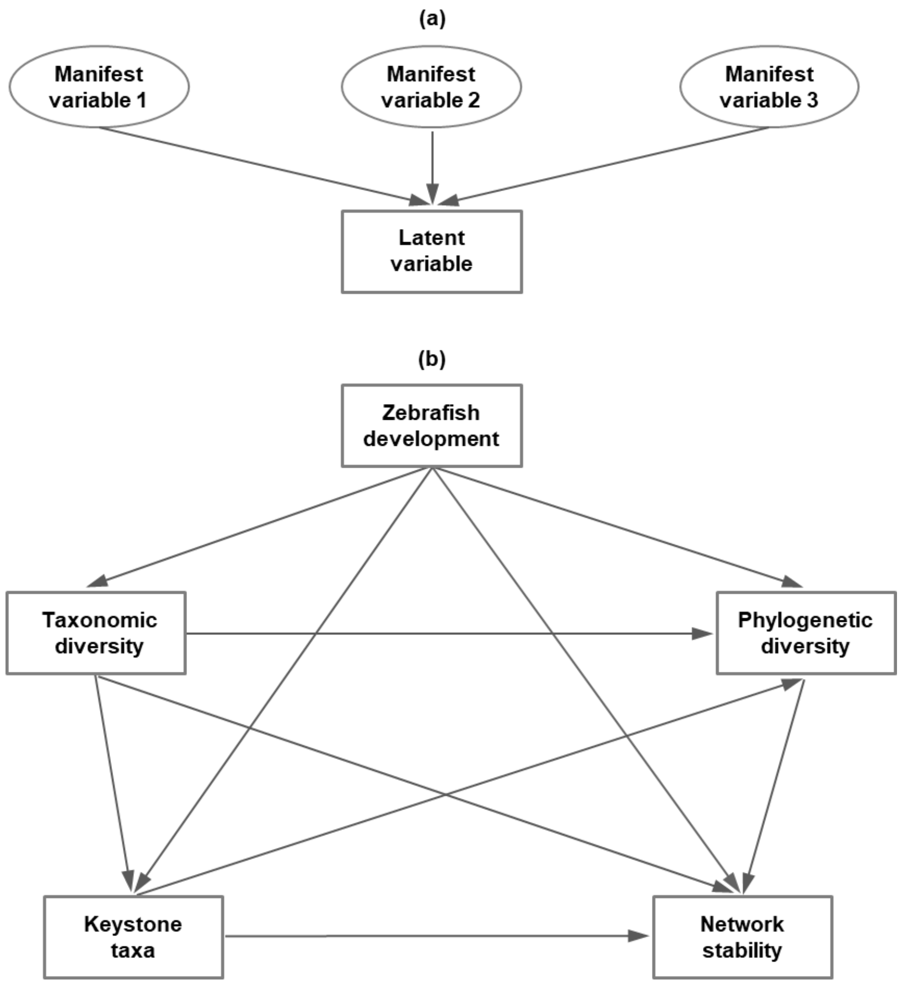

Fig S4 A priori model identifying interrelationships among different factors. (a) A latent variable measured by formative and reflective manifest variables. Latent variables were hypothetical variables that cannot be measured directly and were taken as underlying variables that help explain the association between two or more manifest variables. (b) Path Diagram depicting our model. Zebrafish development box was reflected by stage and days post-hatching (dph); Taxonomic diversity box was reflected by Shannon and Simpson; Phylogenetic diversity box was reflected by phylogenetic distance (PD) and mean pairwise distance (MDP); Keystone taxa box was reflected by connectors and Hubs; Network stability box was reflected by positive (P) and negative cohesion (N).

Table S1 Dissimilarity tests showing the community dissimilarities of gut microbiota across zebrafish development.

Development	MRPP				PERMANOVA			
	Bray-Curtis		Jaccard		Bray-Curtis		Jaccard	
	δ	*p*	*δ*	*p*	*F*	*p*	*F*	*p*
According to sampling points*								
12 dph vs 20 dph	0.551	0.001	0.782	0.001	3.498	0.001	3.122	0.001
20 dph vs 27 dph	0.564	0.001	0.763	0.001	11.024	0.001	5.703	0.001
27 dph vs 42 dph	0.666	0.001	0.776	0.001	17.050	0.001	6.969	0.001
42 dph vs 56 dph	0.615	0.001	0.790	0.001	8.246	0.001	3.214	0.001
56 dph vs 70 dph	0.485	0.001	0.787	0.001	16.676	0.001	5.358	0.001
70 dph vs 98 dph	0.470	0.001	0.780	0.001	20.498	0.001	13.668	0.001
According to developmental stages**								
12-26 vs 27-42 dph	0.679	0.001	0.803	0.001	80.963	0.001	8.877	0.001
12-26 vs 56-98 dph	0.574	0.001	0.815	0.001	24.385	0.001	17.936	0.001
27-42 vs 56-98 dph	0.606	0.001	0.807	0.001	138.053	0.001	13.773	0.001

* Only for those with replicates ≥ 27 per time. ** The stages were divided refer to our previous study [1]. MRPP: multi-response permutation procedure; PERMANOVA: permutational multivariate analysis of variance; dph: day post-hatching.

Table S2 Topological properties of the constructed molecular ecological networks.

Table S3 Taxonomy and classification of potential keystone OTUs as identified by network analysis across zebrafish development.

OTU ID	Average abundance (%)	Keystone type	Phylum	Class	Order	Family	Genus
**12-26 dph**							
OTU91	0.004	Network hubs	Bacteroidetes	Saprospirae	Saprospirales	Chitinophagaceae	*Sediminibacterium*
OTU969	0.019	Network hubs	Proteobacteria	α-Proteobacteria	Rickettsiales	/	/
OTU307	0.019	Network hubs	Proteobacteria	β-Proteobacteria	Methylophilales	Methylophilaceae	*Methylotenera*
OTU1662	0.004	Network hubs	Proteobacteria	γ-Proteobacteria	Enterobacteriales	Enterobacteriaceae	*Serratia*
OTU1204	0.009	Module hubs	Proteobacteria	β-Proteobacteria	Burkholderiales	Oxalobacteraceae	/
OTU204	0.022	Connectors	Planctomycetes	Planctomycetia	Planctomycetales	Planctomycetaceae	*Planctomyces*
OTU295	0.010	Connectors	Planctomycetes	Planctomycetia	Planctomycetales	Planctomycetaceae	*Planctomyces*
OTU141*	0.407	Connectors	Proteobacteria	α-Proteobacteria	Rhizobiales	/	/
OTU335	0.007	Connectors	Proteobacteria	α-Proteobacteria	Rhizobiales	/	/
OTU6695	0.010	Connectors	Proteobacteria	α-Proteobacteria	Rhizobiales	/	/
OTU420	0.015	Connectors	Proteobacteria	α-Proteobacteria	Rhodobacterales	Rhodobacteraceae	*Rhodobacter*
OTU936	0.011	Connectors	Proteobacteria	α-Proteobacteria	Rhodobacterales	Rhodobacteraceae	*Rhodobacter*
OTU720	0.013	Connectors	Proteobacteria	β-Proteobacteria	Burkholderiales	Comamonadaceae	*Comamonas*
OTU303	0.007	Connectors	Proteobacteria	β-Proteobacteria	SC-I-84	/	/
OTU6216	0.003	Connectors	Proteobacteria	γ-Proteobacteria	Enterobacteriales	Enterobacteriaceae	*Plesiomonas*
OTU6832	0.003	Connectors	Proteobacteria	γ-Proteobacteria	Enterobacteriales	Enterobacteriaceae	*Plesiomonas*
OTU7086	0.002	Connectors	Proteobacteria	γ-Proteobacteria	Enterobacteriales	Enterobacteriaceae	*Plesiomonas*
OTU1062	0.021	Connectors	Proteobacteria	γ-Proteobacteria	Legionellales	Coxiellaceae	*Aquicella*
OTU157	0.014	Connectors	Proteobacteria	γ-Proteobacteria	Legionellales	/	/
OTU330	0.010	Connectors	Proteobacteria	γ-Proteobacteria	Pseudomonadales	Pseudomonadaceae	*Pseudomonas*
OTU380	0.024	Connectors	Proteobacteria	γ-Proteobacteria	Pseudomonadales	Pseudomonadaceae	*Pseudomonas*
OTU1050	0.012	Connectors	Proteobacteria	γ-Proteobacteria	Pseudomonadales	Pseudomonadaceae	/
OTU84[#]	0.038	Connectors	Proteobacteria	γ-Proteobacteria	Vibrionales	Vibrionaceae	*Vibrio*
OTU255	0.017	Connectors	Proteobacteria	γ-Proteobacteria	Vibrionales	Vibrionaceae	*Vibrio*
OTU1504	0.012	Connectors	TM6	SJA-4	/	/	/
OTU370	0.019	Connectors	Verrucomicrobia	Verrucomicrobiae	Verrucomicrobiales	Verrucomicrobiaceae	/
OTU476[#]	0.026	Connectors	Verrucomicrobia	Verrucomicrobiae	Verrucomicrobiales	Verrucomicrobiaceae	/

OTU ID	Average abundance	Keystone type	Phylum	Class	Order	Family	Genus
**27-42 dph**	(%)						
OTU52#	0.052	Network hubs	Proteobacteria	α-Proteobacteria	/	/	/
OTU965	0.006	Network hubs	Proteobacteria	γ-Proteobacteria	Vibrionales	Pseudoalteromonadaceae	*Vibrio*
OTU1021#	0.005	Module hubs	Fusobacteria	Fusobacteriia	Fusobacteriales	Fusobacteriaceae	*Cetobacterium*
OTU3089	0.006	Module hubs	Proteobacteria	α-Proteobacteria	Rhizobiales	Phyllobacteriaceae	/
OTU992	0.013	Module hubs	Proteobacteria	β-Proteobacteria	Neisseriales	Neisseriaceae	*Chitinilyticum*
OTU6951	0.003	Module hubs	Proteobacteria	γ-Proteobacteria	Vibrionales	Pseudoalteromonadaceae	/
OTU7032	0.003	Module hubs	Proteobacteria	γ-Proteobacteria	Vibrionales	Pseudoalteromonadaceae	/
OTU934	0.008	Module hubs	Proteobacteria	γ-Proteobacteria	Vibrionales	Pseudoalteromonadaceae	*Vibrio*
OTU860	0.008	Connectors	Acidobacteria	Acidobacteria-6	iii1-15	/	/
OTU298	0.004	Connectors	Actinobacteria	Actinobacteria	Actinomycetales	Mycobacteriaceae	*Mycobacterium*
OTU571#	0.022	Connectors	Bacteroidetes	Flavobacteriia	Flavobacteriales	Flavobacteriaceae	*Flavobacterium*
OTU119#	0.092	Connectors	Planctomycetes	Planctomycetia	Pirellulales	Pirellulaceae	/
OTU366	0.011	Connectors	Planctomycetes	Planctomycetia	Pirellulales	Pirellulaceae	/
OTU436	0.017	Connectors	Planctomycetes	Planctomycetia	Pirellulales	Pirellulaceae	/
OTU465	0.015	Connectors	Planctomycetes	Planctomycetia	Pirellulales	Pirellulaceae	/
OTU717	0.016	Connectors	Planctomycetes	Planctomycetia	Pirellulales	Pirellulaceae	/
OTU772	0.009	Connectors	Proteobacteria	α-Proteobacteria	Rhizobiales	Phyllobacteriaceae	*Mesorhizobium*
OTU451	0.009	Connectors	Proteobacteria	α-Proteobacteria	Rhizobiales	Xanthobacteraceae	/
OTU1625	0.004	Connectors	Proteobacteria	α-Proteobacteria	Rhodobacterales	Rhodobacteraceae	/
OTU6190	0.002	Connectors	Proteobacteria	γ-Proteobacteria	Enterobacteriales	Enterobacteriaceae	*Plesiomonas*
OTU6373	0.003	Connectors	Proteobacteria	γ-Proteobacteria	Enterobacteriales	Enterobacteriaceae	*Plesiomonas*
OTU7084	0.003	Connectors	Proteobacteria	γ-Proteobacteria	Enterobacteriales	Enterobacteriaceae	*Plesiomonas*
OTU563	0.010	Connectors	Proteobacteria	γ-Proteobacteria	Legionellales	Coxiellaceae	/
OTU747	0.006	Connectors	Proteobacteria	γ-Proteobacteria	Legionellales	Legionellaceae	/
OTU810	0.010	Connectors	Proteobacteria	γ-Proteobacteria	Legionellales	/	/
OTU4442	0.003	Connectors	Proteobacteria	γ-Proteobacteria	Vibrionales	Pseudoalteromonadaceae	*Vibrio*
OTU921	0.008	Connectors	Verrucomicrobia	Verrucomicrobiae	Verrucomicrobiales	Verrucomicrobiaceae	*Luteolibacter*

OTU ID	Average abundance	Keystone type	Phylum	Class	Order	Family	Genus
**56-98 dph**	(%)						
OTU551[#]	0.023	Network hubs	Planctomycetes	Planctomycetia	Pirellulales	Pirellulaceae	/
OTU7032	0.002	Network hubs	Proteobacteria	γ-Proteobacteria	Vibrionales	Pseudoalteromonadaceae	/
OTU6121	0.002	Network hubs	Proteobacteria	γ-Proteobacteria	Vibrionales	Pseudoalteromonadaceae	*Vibrio*
OTU7075	0.003	Network hubs	Proteobacteria	γ-Proteobacteria	Vibrionales	Pseudoalteromonadaceae	*Vibrio*
OTU86[#]	0.085	Module hubs	Cyanobacteria	Chloroplast	Stramenopiles	/	/
OTU579	0.014	Module hubs	Proteobacteria	α-Proteobacteria	Rhizobiales	Methylocystaceae	/
OTU440[#]	0.015	Module hubs	Proteobacteria	α-Proteobacteria	Rhizobiales	/	/
OTU481[#]	0.024	Module hubs	Proteobacteria	α-Proteobacteria	Rhodospirillales	Acetobacteraceae	*Roseomonas*
OTU491	0.019	Module hubs	Proteobacteria	γ-Proteobacteria	Legionellales	Coxiellaceae	/
OTU6810	0.003	Module hubs	Proteobacteria	γ-Proteobacteria	Vibrionales	/	/
OTU252[#]	0.030	Module hubs	Proteobacteria	γ-Proteobacteria	Vibrionales	Vibrionaceae	*Vibrio*
OTU367	0.022	Connectors	Chlamydiae	Chlamydiia	Chlamydiales	Parachlamydiaceae	/
OTU963	0.004	Connectors	Nitrospirae	Nitrospira	Nitrospirales	Nitrospiraceae	*Nitrospira*
OTU482	0.008	Connectors	Planctomycetes	Planctomycetia	Gemmatales	Gemmataceae	*Gemmata*
OTU548	0.008	Connectors	Planctomycetes	Planctomycetia	Gemmatales	Gemmataceae	*Gemmata*
OTU578	0.010	Connectors	Planctomycetes	Planctomycetia	Gemmatales	Gemmataceae	*Gemmata*
OTU711	0.006	Connectors	Planctomycetes	Planctomycetia	Gemmatales	Gemmataceae	*Gemmata*
OTU354	0.019	Connectors	Planctomycetes	Planctomycetia	Gemmatales	Gemmataceae	/
OTU424	0.015	Connectors	Planctomycetes	Planctomycetia	Gemmatales	Gemmataceae	/
OTU523	0.009	Connectors	Planctomycetes	Planctomycetia	Pirellulales	Pirellulaceae	*A17*
OTU300	0.026	Connectors	Planctomycetes	Planctomycetia	Pirellulales	Pirellulaceae	*Pirellula*
OTU226[#]	0.052	Connectors	Planctomycetes	Planctomycetia	Pirellulales	Pirellulaceae	/
OTU250[#]	0.025	Connectors	Planctomycetes	Planctomycetia	Pirellulales	Pirellulaceae	/
OTU296	0.024	Connectors	Planctomycetes	Planctomycetia	Pirellulales	Pirellulaceae	/
OTU322	0.024	Connectors	Planctomycetes	Planctomycetia	Pirellulales	Pirellulaceae	/
OTU350	0.018	Connectors	Planctomycetes	Planctomycetia	Pirellulales	Pirellulaceae	/
OTU542	0.010	Connectors	Planctomycetes	Planctomycetia	Pirellulales	Pirellulaceae	/

OTU ID	Average abundance	Keystone type	Phylum	Class	Order	Family	Genus
**56-98 dph (Cont.)**							
OTU632	0.007	Connectors	Planctomycetes	Planctomycetia	Pirellulales	Pirellulaceae	/
OTU687	0.006	Connectors	Planctomycetes	Planctomycetia	Pirellulales	Pirellulaceae	/
OTU366	0.013	Connectors	Planctomycetes	Planctomycetia	Pirellulales	Pirellulaceae	/
OTU677	0.007	Connectors	Planctomycetes	Planctomycetia	Planctomycetales	Planctomycetaceae	*Planctomyces*
OTU685	0.008	Connectors	Planctomycetes	Planctomycetia	Planctomycetales	Planctomycetaceae	*Planctomyces*
OTU857	0.004	Connectors	Planctomycetes	Planctomycetia	Planctomycetales	Planctomycetaceae	*Planctomyces*
OTU988	0.005	Connectors	Planctomycetes	Planctomycetia	Planctomycetales	Planctomycetaceae	*Planctomyces*
OTU295	0.012	Connectors	Planctomycetes	Planctomycetia	Planctomycetales	Planctomycetaceae	*Planctomyces*
OTU947	0.005	Connectors	Proteobacteria	α-Proteobacteria	Rhizobiales	Rhizobiaceae	*Agrobacterium*
OTU1938	0.007	Connectors	Proteobacteria	α-Proteobacteria	Rhizobiales	/	/
OTU858	0.006	Connectors	Proteobacteria	α-Proteobacteria	Rhizobiales	/	/
OTU994	0.008	Connectors	Proteobacteria	α-Proteobacteria	Rhodobacterales	Rhodobacteraceae	*Rhodobacter*
OTU6860	0.002	Connectors	Proteobacteria	α-Proteobacteria	Rhodospirillales	Rhodospirillaceae	*Reyranella*
OTU2731	0.005	Connectors	Proteobacteria	β-Proteobacteria	SC-I-84	/	/
OTU942	0.007	Connectors	Proteobacteria	β-Proteobacteria	SC-I-84	/	/
OTU6151	0.003	Connectors	Proteobacteria	γ-Proteobacteria	Enterobacteriales	Enterobacteriaceae	*Plesiomonas*
OTU6453	0.002	Connectors	Proteobacteria	γ-Proteobacteria	Enterobacteriales	Enterobacteriaceae	*Plesiomonas*
OTU6913	0.002	Connectors	Proteobacteria	γ-Proteobacteria	Enterobacteriales	Enterobacteriaceae	*Plesiomonas*
OTU6916	0.002	Connectors	Proteobacteria	γ-Proteobacteria	Enterobacteriales	Enterobacteriaceae	*Plesiomonas*
OTU6216	0.002	Connectors	Proteobacteria	γ-Proteobacteria	Enterobacteriales	Enterobacteriaceae	*Plesiomonas*
OTU770	0.008	Connectors	Proteobacteria	γ-Proteobacteria	Legionellales	Coxiellaceae	/
OTU526	0.007	Connectors	Proteobacteria	γ-Proteobacteria	Oceanospirillales	Halomonadaceae	*Halomonas*
OTU390	0.014	Connectors	TM6	SJA-4	/	/	/
OTU468	0.014	Connectors	TM6	SJA-4	/	/	/
OTU638	0.007	Connectors	Verrucomicrobia	Opitutae	Cerasicoccales	Cerasicoccaceae	/
OTU765	0.009	Connectors	Verrucomicrobia	Verrucomicrobiae	Verrucomicrobiales	Verrucomicrobiaceae	*Luteolibacter*

Abundant OTU (*), neither abundant nor rare OTU ($^{\#}$), rare OTU (no marks), and "/" indicated unclassified at this classification level.

REFERENCE

1. Xiao F, Zhu W, Yu Y, He Z, Wu B, Wang C, Shu L, Li X, Yin H, Wang J, Juneau P, Zheng X, Wu Y, Li J, Chen X, Hou D, Huang Z, He J, Xu G, Xie L, Huang J, Yan Q. 2021. Host development overwhelms environmental dispersal in governing the ecological succession of zebrafish gut microbiota. npj Biofilms Microbiomes 7:5. https://doi.org/10.1038/s41522-020-00176-2.
